# Fluorimetric Detection of Insulin Misfolding by Probes Derived from Functionalized Fluorene Frameworks

**DOI:** 10.3390/molecules29061196

**Published:** 2024-03-07

**Authors:** Álvaro Sarabia-Vallejo, Ana Molina, Mónica Martínez-Orts, Alice D’Onofrio, Matteo Staderini, Maria Laura Bolognesi, M. Antonia Martín, Ana I. Olives, J. Carlos Menéndez

**Affiliations:** 1 Unidad de Química Orgánica y Farmacéutica, Departamento de Química en Ciencias Farmacéuticas, Facultad de Farmacia, Universidad Complutense, 28040 Madrid, Spain; alsarabi@ucm.es (Á.S.-V.); alicedonofrio@gmail.com (A.D.); mat.staderini@gmail.com (M.S.); 2 Unidad de Química Analítica, Departamento de Química en Ciencias Farmacéuticas, Facultad de Farmacia, Universidad Complutense, 28040 Madrid, Spain; anamolina3006@gmail.com (A.M.); monicifu@gmail.com (M.M.-O.); aiolives@ucm.es (A.I.O.); 3 Department of Pharmacy and Biotechnology, Alma Mater Studiorum—University of Bologna, Via Belmeloro 6, 40126 Bologna, Italy; marialaura.bolognesi@unibo.it

**Keywords:** turn-on fluorescence, fluorescence probes, antioxidants, insulin amyloid conformation, insulin aggregates detection

## Abstract

A group of functionalized fluorene derivatives that are structurally similar to the cellular prion protein ligand *N*,*N*′-(methylenedi-4,1-phenylene)bis [2-(1-pyrrolidinyl)acetamide] (GN8) have been synthesized. These compounds show remarkable native fluorescence due to the fluorene ring. The substituents introduced at positions 2 and 7 of the fluorene moiety are sufficiently flexible to accommodate the beta-conformational folding that develops in amyloidogenic proteins. Changes in the native fluorescence of these fluorene derivatives provide evidence of transformations in the amyloidogenic aggregation processes of insulin. The increase observed in the fluorescence intensity of the sensors in the presence of native insulin or amyloid aggregates suggest their potential use as fluorescence probes for detecting abnormal conformations; therefore, the compounds can be proposed for use as “turn-on” fluorescence sensors. Protein–sensor dissociation constants are in the 5–10 μM range and an intermolecular charge transfer process between the protein and the sensors can be successfully exploited for the sensitive detection of abnormal insulin conformations. The values obtained for the Stern–Volmer quenching constant for compound **4** as a consequence of the sensor–protein interaction are comparable to those obtained for the reference compound GN8. Fluorene derivatives showed good performance in scavenging reactive oxygen species (ROS), and they show antioxidant capacity according to the FRAP and DPPH assays.

## 1. Introduction

Proteins can occur in various conformations that are related to the presence of external stimuli and signaling processes. In most living organisms, including humans, these conformational changes are generally reversible. However, under certain circumstances, thermodynamically favorable conformational changes induce the formation of aberrant proteins that can trigger metabolic imbalances and dangerous pathologies [1]. Abnormal protein folding, and the consequent transformation of harmless alpha conformations of proteins into less soluble beta conformations, leads to the formation of oligomers, protofibrils, fibrils, and aggregates that cannot be eliminated by cell catabolism. These toxic residues are associated with different pathologies in which an abnormal protein is the main trigger of diseases such as diabetes and cancer [2,3,4]. Aberrant conformations of the proteins β-amyloid [5], tau [6], α-synuclein [7], huntingtin [8], fused in sarcoma (FUS) [9], and TDP-43 [10] proteins are responsible for the symptomatology of the most prominent neurodegenerative disorders [11], such as Alzheimer’s, Parkinson’s, and Huntington’s diseases and amyotrophic lateral sclerosis, while infective prion proteins [12] cause irreversible spongiform encephalopathy [13,14].

Insulin is also an amyloidogenic protein [15], and there is evidence that the formation of its amyloid fibrils and amyloid deposits is triggered by mechanical, thermal, and chemical alterations of insulin injectables [16,17]. Localized skin amyloidosis, which is caused by insulin injection, is associated with the deposit of amyloid fibrils which form a subcutaneous mass [18] that appears surrounding the injection site [19,20,21]. In vitro insulin fibrillation [22] requires drug formulations that prevent the formation of abnormal protein conformations during its commercial production and storage by the pharmaceutical industry [23]. The detection of these aggregates in insulin injectables is of great importance, because the presence of insulin fibrils modifies the chemical characteristics and absorption of the injected protein and, ultimately, its bioavailability, thus hampering proper glycemic control in diabetic patients [24,25].

The fluorene ring system has unique luminescence characteristics and its emissive properties are tunable depending on modifications to its chemical structure. Therefore, fluorene derivatives are very interesting as potential photostable fluorescence sensors [26]. Their fluorescence emission is dependent on the polarity of the solvent (solvatochromism) [27] and the presence of certain substituents increases the Stokes shift and enables two-photon excitation. Furthermore, fluorene fluorescence emission can be quenched [28,29] by a variety of small molecules, and the intrinsic fluorescence of proteins can be quenched by fluorene [30]. In addition, the presence of donor-acceptor substituents on the fluorene structure makes intramolecular charge transfer (ICT) processes possible [31]. Finally, fluorene derivatives are generally non-toxic [32].

For these reasons, fluorene derivatives have been proposed for use as fluorescence sensors for studying membrane proteins [33]. A fluorescent sensor having a hybrid structure based on fluorene and Congo red has been proposed for the detection of abnormal beta-like conformations in lysozyme, insulin, and beta-2-microglobulin, and has shown changes in both absorption spectra and fluorescence intensity in the presence of all these proteins [34]. Polyfluorene probes carrying cationic groups are capable of interacting with the negatively charged residues of proteins through ionic bonds while emitting fluorescence as a result of the aggregation process (aggregation-induced emission) [35]. In addition, conjugated polymers based on fluorene and other fluorophores capable of fluorescence resonance energy transfer (FRET) are very useful as colorimetric and fluorimetric sensors for the point-of-care detection of serum albumin [36].

Several in vitro studies have shown that fluorene derivatives with small structural modifications in the fluorene ring can play a key role in inhibiting fibrillation and aggregation of human beta-amyloid protein, indicating potential therapeutic applications for neurodegenerative diseases such as Alzheimer’s. Bifunctional nitroxide-bromofluorene compounds successfully inhibited the oligomerization and aggregation of the human amyloid-β-peptide Aβ (1–40) [37,38], while compounds comprising conjugated oligo(fluorene-co-phenylene) fused with 1,8-naphthalimide cores were able to prevent the formation of β-amyloid protein aggregates [39]. On the other hand, conjugated fluorene polymers are able to interact with α-synuclein and accelerate its aggregation, the results suggesting that the hydrophobicity of the polymer facilitates its association with the protein framework [40]. Multitarget drugs based on hydroxyethylamino and fluorene fragments were studied for the treatment of Alzheimer’s disease, and some of the derivatives showed both selective butyrylcholinesterase inhibitory activity related to the structure of hydroxyethylamino fragment and adequate inhibition of β-amyloid aggregation due to the presence of the fluorene ring [41]. A number of 2-amino-7-bromofluorene derivatives demonstrated good free radical trapping capacity and anti-inflammatory properties in brain endothelial tissue cells, providing a rationale for their potential application in neurodegenerative diseases [42,43].

The study of some amyloid proteins and peptides implicated in cellular toxicity (prion proteins, α-synuclein, Aβ, insulin, etc.) has suggested that only short segments of the full sequence are involved in the conversion of the protein to the amyloid state [44]. These segments, which can be as short as six amino acids and have a high propensity to exist in β-sheet conformation, are known as aggregation-prone segments (APRs), self-recognition elements (SREs), or hot spots (HSs) and have common structural features for all proteins, such as high hydrophobicity and low net charge. It can be thus expected that a compound interacting with the hot spots of one of these proteins may be able to extend this behavior to others.

**Figure 1:**
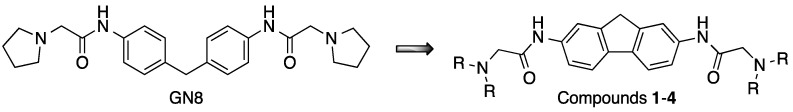
Rationale for the design of compounds **1**–**4**.

In an effort to identify new chemotypes for the labeling of insulin fibrillar species, we focused our attention on a small library of functionalized fluorene derivatives **1**–**4** (Figure 1). These compounds are rigid analogues of GN8, which is known to stabilize the conformation of the cellular prion protein (PrP^C^) by interacting with its hot spots and inhibiting its transformation into the toxic scrapie form (PrP^Sc^) [45,46]. The choice of fluorene as the central core of our structures was dictated by its high native fluorescence, which allows for its sensitive detection by fluorimetry. Furthermore, we have previously shown the usefulness of the GN8 rigidification approach by showing that carbazole-derived GN8 analogues retain their activity on prion proteins [47]. Thus, the aim of the present work is to study the application of fluorene derivatives **1**–**4** as sensitive fluorescence probes for the detection of aggregates of insulin amyloid fibrils in pharmaceutical samples via fluorescence events such as the quenching or enhancement of signals.

## 2. Results and Discussion

### 2.1. Synthesis

**Scheme 1:**
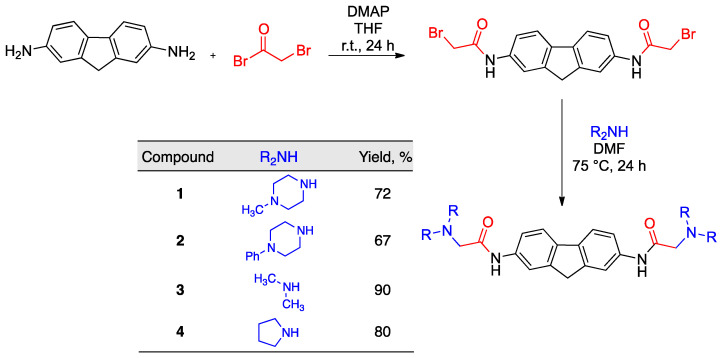
Synthesis of fluorene derivatives **1**–**4**.

The fluorene derivatives were obtained by transforming commercially available 9*H*-fluorene-2,7-diamine into a bis-halide by coupling with bromoacetyl bromide in the presence of 4-dimethylaminopyridine (DMAP) in THF. This intermediate was treated with the suitable secondary amines to afford compounds **1**–**4** in good yields by the double nucleophilic displacement of bromide anions by *N*-nucleophiles (Scheme 1).

### 2.2. Spectrophotometric and Spectrofluorimetric Study of Fluorene Derivatives

The UV–Visible absorption spectra of the fluorene derivatives **1**–**4** in ethanol show absorption in the 200–350 nm region, depending on the compound’s structure. Thus, compounds **1** and **2** present maxima close to 250 nm and 290 nm, which are shifted to 305 and 325 nm for **3** and **4**. The UV–Vis absorption spectra are shown in Figure S1 (Supporting Information), while Table S1 summarizes their molar absorptivities, calculated from the absorbance values at the maximum absorption wavelengths. These molar absorptivity values are important to establish an accurate concentration of the compounds with regard to the molar concentrations of proteins and protein fibrils. The influence of substituents on the fluorene moiety was found to be more significant than the polarity of the environment, which does not affect the absorption maximum wavelength (Table S2). 

It is well known that the fluorescence emission spectrum of fluorene shows an emission band (290–370 nm) with maxima at 305 and 325 nm (λ_ex_ 250–260 nm) when dissolved in non-polar and polar organic solvents such as alkanes and ethanol, respectively. The native fluorescence of fluorene is sensitive to structural manipulation, since the introduction of a methyl group at C-1 causes the appearance of a new emission maximum at 315 nm in addition to those mentioned above. Moreover, the fluorescence emission of substituted fluorene derivatives is sensitive to the environment, with shifts in the emission maxima being observed as a consequence of changes in solvent polarity. This is the case for 9,9-dimethyl-7-(10*H*-phenoxazine-10-yl)-9*H*-fluorene-2-carbonitrile, where the fluorescence maximum shifts from 433 nm in cyclohexane to 620 nm in acetonitrile, which is accompanied by a significant decrease in fluorescence emission intensity in the case of higher polarity solvents such as acetonitrile [27]. Conjugation broadens the emission spectrum, as observed on 1,2-benzofluorene (330–450 nm, with maxima at 345 and 365 nm) [48,49]. Structural modification based on donor–acceptor effects has important consequences as it shifts the emission maxima towards the red, thus enabling intramolecular charge transfer (ICT) phenomena [32].

**Figure 2:**
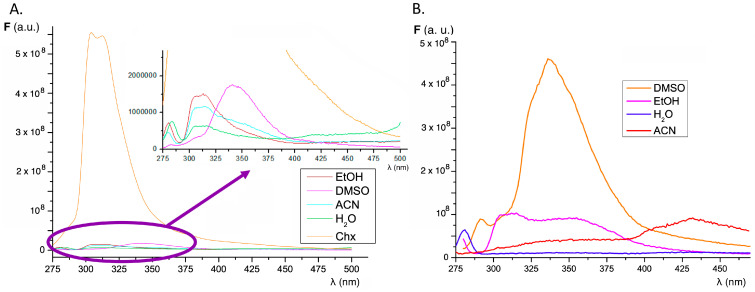
Effect of solvent polarity on the fluorescence emission spectra of compound **1** (**A**) and compound **2** (**B**) at maximum excitation wavelength corresponding to each solvent (Table S3). The inset shows the expanded view of the spectra in order to compare the magnitude of the fluorescence emission intensity in different solvents.

The compounds under study were designed on the basis that ring substituents shift the emission maxima and, at the same time, are sufficiently flexible to bind to the folds of the anomalous conformations of proteins [50]. Figure 2 shows the fluorescence emission spectra of compounds **1** and **2** in the different solvents studied. For both compounds, the highest fluorescence emission intensity is observed in cyclohexane and DMSO solution, while a very low emission intensity is observed in aqueous media. In addition, the emission band shifts from 305–315 nm in cyclohexane to around 350 nm in DMSO, while a very weak emission in the region of 400–450 nm is observed in acetonitrile, suggesting intramolecular charge transfer processes between the fluorene substituents and the base fluorophore. The fluorescence emission maxima in various solvents are summarized in Table S3.

### 2.3. Interaction with Insulin and Insulin Fibrils

**Figure 3:**
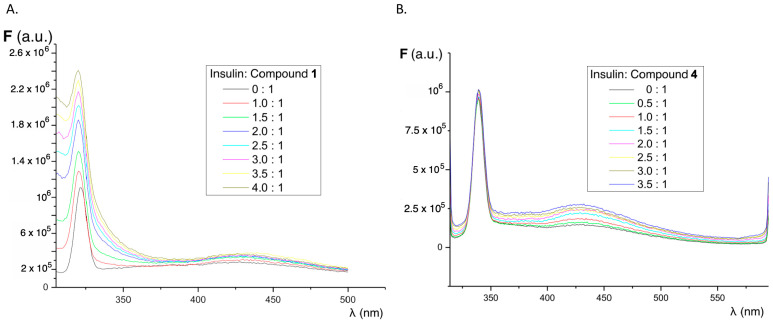
Effect of the addition of increasing volumes of native insulin on the fluorescence emission spectra of compound **1** at λ_ex_ = 289 nm (**A**) and compound **4** at λ_ex_ = 304 nm (**B**). The protein:sensor molar ratio is shown in the inset.

The influence of increasing concentrations of insulin on the fluorescence of compounds **1**–**4** was studied in order to determine whether the interaction with native insulin (alpha-helix conformation) causes changes in the fluorescence of our sensors. Parallel experiments were performed using fluorene and thioflavin T as references. As shown in Figure 3, the presence of increasing concentrations of native insulin results in only a weak increase in the fluorescence emission intensity of all compounds. Fluorescence spectra show a peak around 325–350 nm due to the Raman effect. It is important to note that, in the presence of protein, the fluorescence emission of the sensors is shifted to a charge transfer emission band with maxima in the region of 430 nm. This emission maximum is observed only in polar organic solvents (ethanol and acetonitrile) and it indicates that the sensor is in a lower polarity environment, such as a hydrophobic pocket in the protein. Indeed, in buffered aqueous solution, the fluorescence emission is negligible in the same experimental conditions. In the case of fluorene, neither increases in the fluorescence intensity nor shifts in the emission properties were observed (Figure S2A). For thioflavin T, there is a weak fluorescence emission band increase in the presence of growing concentrations of native insulin, but the most significant peak corresponds to the Raman effect (Figure S2B).

**Figure 4:**
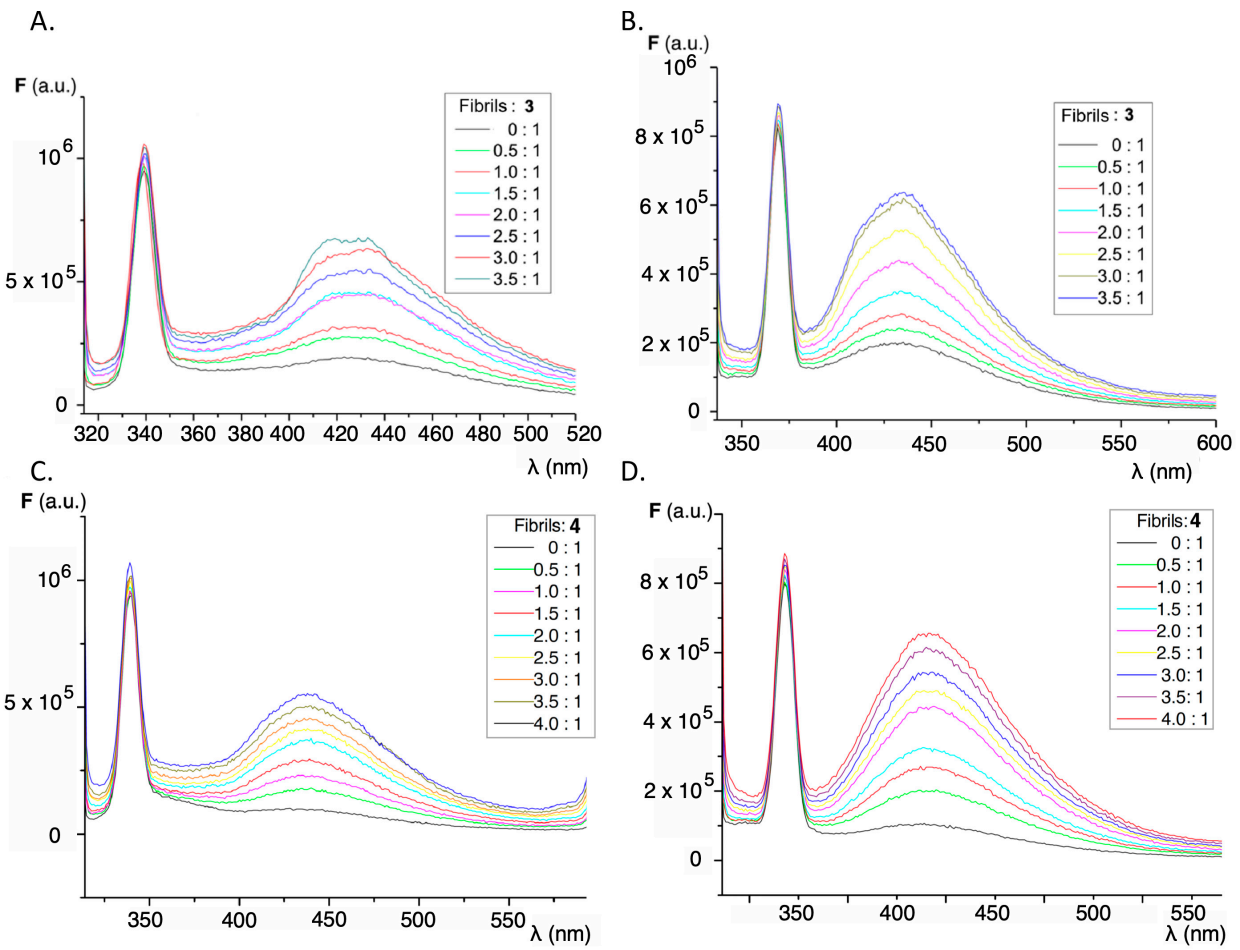
Effect of the additions of increasing volumes of insulin fibrils/aggregates on the fluorescence emission spectra of compound **3**: (**A**) at λ_ex_ = 304 nm; (**B**) at λ_ex_ = 327 nm, employing method A for protein aggregation. Same experiments for compound **4**: (**C**) at λ_ex_ = 304 nm; (**D**) at λ_ex_ = 327 nm, employing method A for protein aggregation. The protein:sensor molar ratios are in the inset. The peaks at 340 (**A**,**C**) and 370 nm (**B**,**D**) correspond to Raman dispersion.

**Figure 5:**
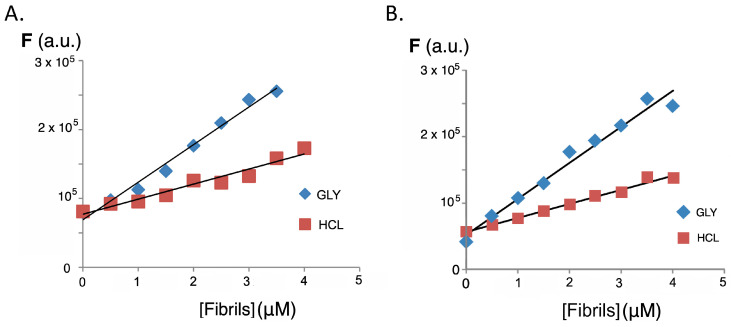
(**A**) Comparison of the increase in the fluorescence intensity of compound **3** (λ_ex_ = 327; λ_em_ = 435 nm) when employing method A (GLY) and method B (HCL) for inducing insulin β-amyloid protein aggregation. (**B**) Same experiments for compound **4**.

High temperature and strongly acidic pH are known to be the main determinants in the formation of insulin amyloid aggregates, and the combination of both conditions was used as the basis for two methods for the formation of the abnormal insulin fibril conformations (see methods A and B in Section 3.4). The main difference between both methods for the preparation of fibrils/aggregates lies in the centrifugation step used to remove the non-soluble forms of the protein used in method A (GLY), which incorporates glycine in the buffer, while only hydrochloric acid was used in method B (HCL). The formation of insulin amyloid aggregates was tested by titrating a solution of thioflavin T, well-known as gold standard for the formation of insulin fibrils [51]. The characteristic emission band of thioflavin bound to insulin fibrils can be seen from the characteristic fluorescence appearing at 485–490 nm when excited at λ_ex_ 440 nm (Figure S3). The observed increase in fluorescence intensity for the same concentration of fibrils or the same protein concentration is higher for method A (GLY), as can be appreciated in Figure S4. When the titrations were carried out with insulin amyloid fibrils, a significant increase in the fluorescence intensity of the fluorene derivatives was observed. This increase in fluorescence is dependent on the excitation wavelength used and also on the procedure employed in the formation of the insulin amyloid fibrils. Figure 4 shows the behavior for compounds **3** and **4**, and Figure 5 compares the increase in fluorescence intensity according to the fibril formation methodology. As with thioflavin, method A produces a significantly greater fluorescence enhancement and higher slopes than those observed in the case of method B.

**Figure 6:**
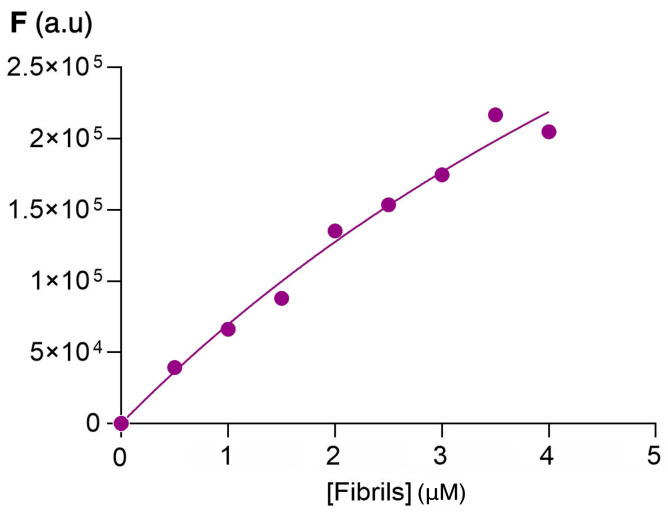
Protein fibrils–sensor binding assay. Fluorescence plots for determination of the dissociation constant of compound **4** and fibrils of insulin produced by method A. Dots correspond to average experimental data and the line corresponds to the mathematical fitting.

Considering the increase in fluorescence intensity in the functionalized fluorene derivatives for the emission band appearing at 400–450 nm, the dissociation constants (*K*_d_) were determined using the Michaelis–Menten kinetics model as described in Section 3.7. Figure 6 shows the variation of fluorescence emission with increasing amounts of insulin amyloid fibrils, while Table 1 summarizes the values of the dissociation constants obtained compared with the dissociation constant of thioflavin in the same conditions. These values were obtained assuming only one binding site on the protein and are in the same range as those obtained for thioflavin T (Table 1) and other sensors for insulin fibrils [52,53]. The relatively high dissociation constant values show that the sensor–protein interaction is reversible, a key feature in the discovery of new sensor molecules.

Given that the charge transfer processes involved in fluorescence emission are highly dependent on the molecular environment [54], and taking into account the overlap between the emission bands of the protein (insulin) and our fluorene-derived sensors, the fluorescent behavior of these compounds was studied by keeping the protein concentration constant and varying the sensor concentration. When excited at the excitation wavelength of most proteins, namely in the 250–270 nm region, fluorescence can be observed in the 300–320 nm region, mainly due to the fluorescence of the aromatic amino acids tryptophan, phenylalanine, and tyrosine. Fluorene-functionalized derivatives also show an absorption and excitation maximum of fluorescence in the 250–300 nm region. Therefore, keeping constant the concentration of insulin amyloid fibrils obtained by method A, and titrating with increasing concentrations of the fluorene derivatives, a gradual decrease in the fluorescence emission intensity of the protein is observed at its maximum of 300–310 nm (λ_ex_ = 275 nm). As this band decreases, there is an increase in the emission band at 430–450 nm (corresponding to the fluorene derivatives) due to the intramolecular charge transfer process favored in the environment of the beta folding of insulin. This behavior can be observed in Figure S5 and is clearly different from the one detected in native fluorene. Since the fluorene core does not possess other potential functional groups that could receive this energy, even in the presence of increasing concentrations of insulin amyloid fibrils no intramolecular charge transfer phenomenon was observed (Figure S6). In this case, the only consequence of the presence of insulin is a decrease in the fluorescence emission characteristics of fluorene.

**Figure 7:**
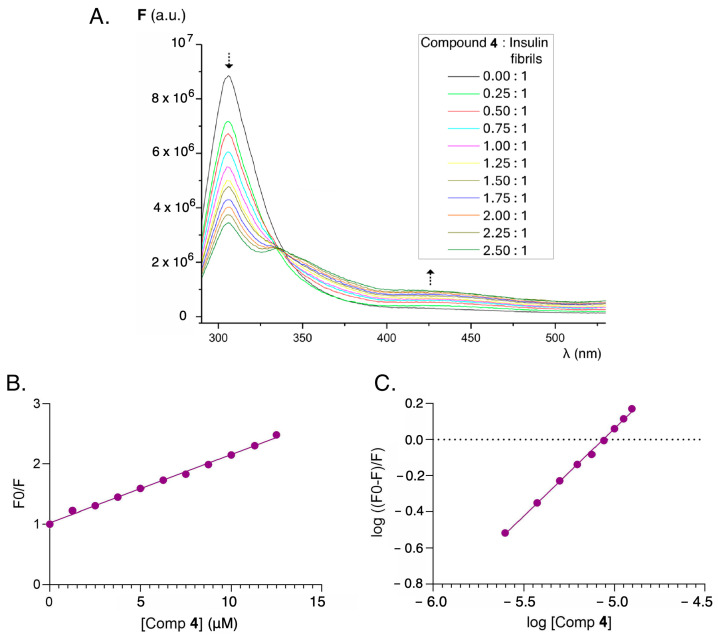
(**A**) Fluorescence emission spectra (λ_ex_ = 275 nm) of amyloid insulin in the presence of increasing concentrations of compound **4**. (**B**) Stern–Volmer plot for the quenching emission (λ_em_ = 305 nm) of amyloid insulin induced by compound **4**. (**C**) Double logarithmic plot for describing the fluorescence quenching (λ_em_ = 305 nm) of amyloid insulin induced by compound **4**. Amyloid insulin fibrils obtained according to method A. Concentration of insulin fibrils 5 × 10^−6^ M. The concentration of compound **4** varies from 0 to 12.5 × 10^−6^ M.

In many cases it is possible to establish the interaction of small organic molecules with biomolecules on the basis of fluorescence-quenching phenomena arising from the protein binding to the drug. Thus, the decrease in the fluorescence intensity of human serum albumin in the presence of increasing concentrations of riboflavin is attributable to the interaction between the vitamin and the protein, with a decrease in the characteristic emission band of the protein in the presence of increasing concentrations of riboflavin [55]. This behavior has also been described in thioflavin and its interaction with human and bovine serum albumin [56]. Thus, when insulin amyloid fibril solutions are titrated with thioflavin T at increasing concentrations, the fluorescence intensity at 305 nm decreases while the emission intensity increases at the characteristic maximum of thioflavin as thioflavin concentrations increase and become incorporated into the beta-folding of insulin. An isoemissive point is observed at 347 nm, which corroborates the protein–sensor interaction (Figure S7). An analogous damping phenomenon, but of different magnitude, can be seen in the compounds studied, as well as in the reference compound GN8 [45,46] (Figure S10). The overlapping emission spectra of protein (λ_em_ = 305 nm) and excitation spectra of the fluorene derivatives ((λ_ex_ = 300–310 nm) lead to an intermolecular resonance energy transfer effect (RET), giving rise to a quenching effect of the protein fluorescence by the sensors, particularly in compound **4**, that can be exploited for the sensitive detection of abnormal insulin conformations. By way of example, the behavior found in the functionalized fluorene derivatives is shown for the case of compound **4** in Figure 7.

Taking into account the fluorescence quenching effect, the fluorescence values were processed at different concentrations of the quencher (fluorene derivatives), and the Stern–Volmer constant and the binding constant were determined according to the method described in Section 3.8 (Figure S8, Figure S9, Figure S11 and Figure S12) [57]. In the case of compound **4**, the binding constant obtained was higher than those of GN8 and thioflavin T (Table 2). This means that this compound can be considered as an attractive sensor for detecting anomalous conformations of insulin which may potentially inhibit the abnormal folding of proteins.

Compounds **1–3** also behave as quenchers of the fluorescence of amyloid insulin fibrils, but the experimental values could not be adjusted to the models considered for the quantitative evaluation of the process.

### 2.4. Antioxidant Capacity

**Figure 8:**
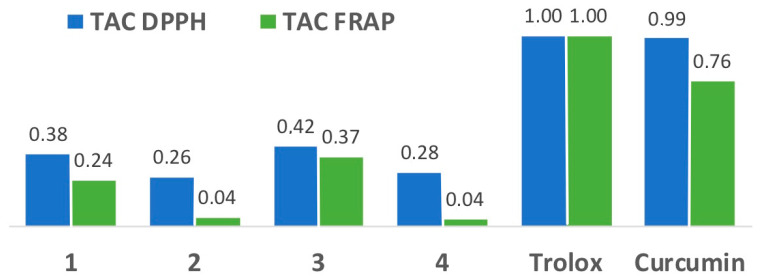
Total antioxidant capacity (TAC) of compounds **1**–**4** as compared to Trolox and curcumin.

Chronic high glucose levels found in diabetes are associated with an increase in reactive oxygen species (ROS). Therefore, we determined the antioxidant capacity of the fluorene derivatives **1**–**4** using two complementary methods, namely the DPPH and FRAP assays. The former evaluates the hydrogen atom transfer capacity to reduce an odd electron, while in the latter assay the antioxidant capacity is related to electron transfer reactions. The results obtained are summarized in Figure 8, and show that all compounds have an appreciable antioxidant capacity, especially in the DPPH assay, although it is less than that of the reference antioxidants Trolox and curcumin, with compounds **1** and **3** having the highest activity. Increased ROS levels are an important driver of diabetic complications since they can activate apoptosis in pancreatic β-cells [58], and therefore the antioxidant capacity can have some therapeutic value. It is relevant to mention that our compounds show higher antioxidant capacity than other fluorene derivatives studied in the literature [59].

## 3. Materials and Methods

### 3.1. General Experimental Information

NMR spectra were registered with a Bruker Avance 250 spectrometer (250 MHz for ^1^H, 63 MHz for ^13^C) maintained by the Unidad de Resonancia Magnética Nuclear, UCM. Infrared spectra were obtained by preparing KBr disks for each compound and registering their spectra with a Perkin Elmer Paragon 1000 FT-IR spectrophotometer. Melting points were taken with a Reichert 723 hot stage microscope. CHN combustion elemental analyses were performed by the Unidad de Microanálisis Elemental, UCM.

UV–VIS absorption spectra were registered with a Cary 60 spectrophotometer (Agilent, Santa Clara, CA, USA) using Cary WinUV software (vs 5.1.0.1016). Excitation and emission spectra and fluorescence measurements at a fixed wavelength were registered with a FluoroMax-4 spectrofluorometer (Horiba-Jobin Yvon, Kyoto, Japan) with excitation and emission slits set at 5 nm, using FluorEssence 2.1 software. Quartz cells with a 1 cm path length were employed throughout. UV–VIS absorption measurements at fixed wavelengths were taken with transparent polystyrene plates containing 96 wells using a FLUOstar Omega microplate reader (BMG Labtech, Ortenberg, Germany) equipped with Omega Control 5.70 R2 software. The reaction media used for the FRAP experiments were incubated in an Accublock D-1301 dry bath (Labnet, Madrid, Spain).

Synthetic reagents, thioflavin, and a 10 mg/mL human insulin solution (MW = 5800) were purchased from Merck-Sigma-Aldrich (Burlington, MA, USA). Analytical-grade or ACS-grade sodium phosphate, glycine, and hydrochloric acid were obtained from Panreac (Castellar del Vallès, Spain). Synthetic, analytical, or spectroscopic-grade solvents were purchased from SDS (Messia-sur-Sorne, France) or Panreac. Ultrapure water was generated using a Milli-Q Direct 8 system (Millipore, Molsheim, France).

### 3.2. Synthesis of Fluorene Derivatives. General Procedure

A solution of fluorene-2,7-diamine (300 mg, 1.53 mmol) and 4-dimethylaminopyridine (460 mg, 3.8 mmol) in dry tetrahydrofuran (15 mL), under an argon atmosphere, was treated dropwise with bromoacetyl bromide (0.33 mL, 3.8 mmol). The solution was stirred at room temperature (24 h), and then 0.1 M aqueous hydrochloric acid (15 mL) was added. The resulting solution was stirred at room temperature for 30 min and filtered, and the solid was washed with water until it no longer gave an acidic reaction on pH paper. It was then dried under vacuum to give *N*,*N*′-(9*H*-fluorene-2,7-diyl)bis(2-bromoacetamide) as a pale brown solid (650 mg, 97%) that gave the following spectral characterization data: IR ν*_max_* (KBr): 1683, 3257 cm^−1^. ^1^H NMR (250 MHz, DMSO) δ 3.94 (s, 2H), 4.08 (s, 4H), 7.55 (dd, *J* = 8.2, 2.5 Hz, 2H), 7.80 (d, *J* = 8.2 Hz, 2H), 7.91 (s, 2H), 10.49 (br s, 2H) ppm. ^13^C-NMR (63 MHz, d_6_-DMSO) δ 31.4 (2C), 37.5, 116.9 (2C), 118.9 (2C), 120.7 (2C), 137.6 (2C), 138.1 (2C), 144.6 (2C), 165.5 (2C) ppm.

To a solution of the crude dibromide described above (200 mg, 0.45 mmol) in dry dimethylformamide (2 mL) at 75 °C, under an argon atmosphere, two equivalents of the suitable secondary amine was added dropwise. The reaction mixture was stirred for 24 h at 75 °C, and then it was allowed to cool to room temperature and treated with 10% aqueous potassium carbonate (5 mL). The precipitate that was formed was filtered under reduced pressure, washed with diethyl ether (3 × 1 mL), and dried to provide compounds **1**–**4**.

#### 3.2.1. *N*,*N*′-(9*H*-Fluorene-2,7-diyl)bis(2-(4-methylpiperazin-1-yl)acetamide) (**1**)

The first compound was prepared from *N*,*N*′-(9*H*-fluorene-2,7-diyl)bis(2-bromoacetamide) (200 mg, 0.45 mmol) and 4-methylpiperazine (90.1 mg, 0.90 mmol), yielding 154.3 mg (72%) as a pale brown solid. [Found: C, 67.92; H, 7.36; N, 17.45. C_27_H_36_N_6_O_2_ requires C 68.04, H 7.61, N 17.63]; Mp, 139–142 °C. IR ν*_max_* (KBr): 1472, 1683, 3278 cm^−1^. ^1^H NMR (300 MHz, CDCl_3_) δ 2.33 (s, 6H), 2.52 (s, 8H), 2.67 (s, 8H), 3.16 (s, 4H), 3.89 (s, 2H), 7.44 (dd, *J* = 8.3, 2.1 Hz, 2H), 7.66 (d, *J* = 8.2 Hz, 2H), 7.89 (s, 2H), 9.20 (s, 2H), ppm. ^13^C NMR (75 MHz, CDCl_3_) δ 37.2, 46.1 (2C), 53.6 (4C), 55.4 (4C), 62.0 (2C), 116.5 (2C), 118.3 (2C), 119.9 (2C), 136.3 (2C), 137.8 (2C), 144.4 (2C), 168.4 (2C) ppm.

#### 3.2.2. *N*,*N*′-(9*H*-Fluorene-2,7-diyl)bis(2-(4-phenylpiperazin-1-yl)acetamide) (**2**)

The second compound was prepared from *N*,*N*′-(9*H*-fluorene-2,7-diyl)bis(2-bromoacetamide) (0.2 g, 0.45 mmol) and 4-phenylpiperazine (146 mg, 0,90 mmol), yielding 181.1 mg (67%) as a pale brown solid. [Found: C, 73.59; H, 6.66; N, 13.82. C_37_H_40_N_6_O_2_ requires C 73.97, H 6.71, N 13.99]; Mp, 224–227 °C. IR ν*_max_* (KBr): 1681 2825, 3291 cm^−1^. ^1^H NMR (250 MHz, CDCl_3_) δ 2.87 − 2.85 (m, 8H), 3.27 (s, 4H), 3.34 − 3.31 (m, 8H), 3.93 (s, 2H), 7.01 − 6.90 (m, 6H), 7.36 − 7.29 (m, 4H), 7.48 (d, *J* = 8.25, 2H), 7.70 (d, *J* = 8.2 Hz, 2H), 7.93 (s, 2H), 9.27 (s, 2H) ppm. ^13^C-NMR (63 MHz, CDCl_3_) δ 37.6, 49.9 (4C), 54.0 (4C), 62.4 (2C), 116.7 (4C), 116.8 (2C), 118.6 (2C), 120.2 (2C), 120.6 (2C), 129.7 (4C), 136.5 (2C), 138.1 (2C), 144.7 (2C), 151.4 (2C), 168.4 (2C) ppm.

#### 3.2.3. *N*,*N*′-(9*H*-Fluorene-2,7-diyl)bis(dimethylamino)acetamide (**3**)

The third compound was prepared from *N*,*N*′-(9*H*-fluorene-2,7-diyl)bis(2-bromoacetamide) (0.2 g, 0.45 mmol) and 40% aqueous dimethylamine (101 µL, 0.90 mmol), yielding 148.4 mg (90%) as a pale brown solid. [Found: C, 68.69; H, 6.98; N, 15.18. C_21_H_26_N_4_O_2_ requires C 68.83, H 7.15, N 15.29]; Mp, 204–207 °C. IR ν*_max_* (KBr): 1518, 1682, 3258 cm^−1^. ^1^H NMR (250 MHz, DMSO) δ 2.50 (s, 6H), 3.10 (s, 4H), 3.88 (s, 2H), 7.61 (d, *J* = 8.2, 2H), 7.74 (d, *J* = 8.2 Hz, 2H), 7.96 (s, 2H), 9.78 (br s, 2H) ppm. ^13^C-NMR (63 MHz, DMSO) δ 37.0, 45.7 (4C), 63.7 (2C), 116.6 (2C), 118.6 (2C), 119.8 (2C), 136.7 (2C), 137.5 (2C), 143.8 (2C), 168.8 (2C) ppm.

#### 3.2.4. *N*,*N*′-(9*H*-Fluorene-2,7-diyl)bis(2-(pyrrolidin-1-yl)acetamide) (**4**)

The fourth compound was prepared from *N*,*N*′-(9*H*-fluorene-2,7-diyl)bis(2-bromoacetamide) (0.2 g, 0.45 mmol) and pyrrolidine (76 µL, 0.90 mmol), yielding 150.7 mg (80%) as a yellow solid. [Found: C, 71.51; H, 7.15; N, 13.23. C_21_H_26_N_4_O_2_ requires C 71.74, H 7.22, N 13.39]; Mp, 174–177 °C. IR ν*_max_* (KBr): 1475, 1689, 3324 cm^−1^. ^1^H NMR (250 MHz, DMSO) δ 1.80 − 1.78 (m, 8H), 2.63 − 2.59 (m, 8H), 3.26 (s, 4H), 3.86 (s, 2H), 7.59 (d, *J* = 8.2, 2H), 7.74 (d, *J* = 8.2 Hz, 2H), 9.77 (s, 2H), 7.94 (s, 2H) ppm. ^13^C-NMR (63 MHz, DMSO) δ 23.8 (4C), 36.9, 54.1 (4C), 59.9 (2C), 116.6 (2C), 118.6 (2C), 119.8 (2C), 136.7 (2C), 137.5 (2C), 143.8 (2C), 168.9 (2C) ppm.

### 3.3. Synthesis of GN8

This compound was obtained using a modified literature protocol [45]. A solution of 4,4′-methylenedibenzenamine (202 mg, 1.02 mmol), 2-bromoacetic acid (284 mg, 2.06 mmol), 1-ethyl-3-(3-dimethylaminopropyl)carbodiimide (382 mg, 2.00 mmol), and triethylamine (147 µL, 2.0 mmol) in dry DMF (5 mL) was stirred under argon for 2 h. The reaction mixture was diluted with ethyl acetate (15 mL) and the organic layer was washed with 1 M aqueous HCl (2 × 5 mL) and 5% aqueous sodium bicarbonate (2 × 5 mL) and dried over anhydrous sodium sulfate. The solvent was evaporated to give *N*,*N*′-[4,4′-methylenebis(4,1-phenylene)]bis(2-bromoacetamide) (200 mg, 46%) as a pale brown solid. A solution of this dibromide (100 mg, 0.23 mmol) and pyrrolidine (28 µL, 0.46 mmol) in dry dimethylformamide (2 mL) was stirred for 24 h at 75 °C under an argon atmosphere. The mixture was allowed to cool to room temperature and 10% aqueous potassium carbonate (2 mL) was added. The precipitate thus formed was filtered, washed with ethyl ether (2 × 3 mL) and dried under reduced pressure to give GN8 (62 mg, 64%) as a pale brown solid. [Found: C, 71.16; H, 7.72; N, 13.35. C_25_H_32_N_4_O_2_ requires C 71.40, H 7.67, N 13.32]; Mp, 144–147 °C. IR ν*_max_* (KBr): 814, 1513, 1664, 1681, 2799, 2954, 3257 cm^−1^. ^1^H NMR (250 MHz, d_6_-DMSO) δ 1.76 (br s, 8H), 2.64 (br s, 8H), 3.30 (s, 4H), 3.84 (s, 2H), 7.13 (d, *J* = 8.4 Hz, 4H), 7.54 (d, *J* = 8.4 Hz, 4H), 9.70 (br s, 2H) ppm. ^13^C-NMR (63 MHz, DMSO) δ 24.2 (4C), 41.0, 54.6 (4C), 60.1 (2C), 120.4 (4C), 129.6 (4C), 137.3 (2C), 137.5 (2C), 169.0 (2C) ppm.

### 3.4. Preparation of Amyloid β Fibrils from Human Insulin

Method A: A 1.65 mM solution of human insulin in 50 mM glycine-HCl buffer (pH = 2.2) was heated in a water bath at 65 °C for 18 h. The fibrils were separated from the non-solubilized protein by centrifugation (5000 rpm × 3 min) [60]. A 1.0 × 10^−5^ M solution of the insulin amyloid fibrils in water was prepared and its concentration was checked spectrophotometrically at λ = 276 nm using the molar absorptivity value ε = 6191.5 M^−1^cm^−1^ [61].

Method B: A 0.50 mM solution of human insulin in 25 mM aqueous HCl (pH = 2.0–2.2) was heated in a water bath at 65 °C for 18 h [62]. An aliquot of this solution was taken for preparing an aqueous solution of insulin amyloid fibrils (approx. 1.0 × 10^−5^ M) and the actual concentration of insulin was verified by spectrophotometry (λ = 276 nm; ε = 6191.5 M^−1^cm^−1^).

### 3.5. Spectrophotometric and Spectrofluorimetric Study of Functionalized Fluorene Derivatives

The compounds were characterized spectrophotometrically in order to accurately establish the sensor–protein molar ratio. An exact amount of each of the compounds studied was weighed and dissolved in an appropriate volume of ethanol to produce solutions of concentration 1.0 × 10^−3^ M. To achieve complete dissolution, it was necessary to keep the compounds in an ultrasonic bath for at least 30 min, and then the solutions thus obtained were left to stand for an additional 2 h. Aliquots were then taken and ethanol solutions of concentrations ranging between 1.0 × 10^−5^ M and 1.0 × 10^−4^ M were prepared. UV–Vis absorption spectra (200–500 nm) were obtained and the molar absorptivities of each compound were determined at the maximum absorption wavelength.

For the spectrofluorimetric characterization of the fluorene derivatives, appropriate aliquots of the ethanolic solution (1.0 × 10^−4^ M) were taken and solutions of 1.0 × 10^−6^ M concentration were prepared. Then, the excitation and fluorescence emission spectra were obtained.

The influence of solvent polarity on the spectroscopic properties of fluorene derivatives was studied both spectrophotometrically and spectrofluorimetrically. For this purpose, from the 1.0 × 10^−3^ M stock solution, 1.0 × 10^−5^ M solutions were prepared in cyclohexane, ethanol, acetonitrile, and water. Absorption spectra were then recorded between 200 and 500 nm. From the previously prepared solutions in the different solvents, solutions of concentration 1.0 × 10^−6^ M, and in some cases 5.0 × 10^−7^ M, were prepared in cyclohexane, ethanol, acetonitrile, and water. DMSO solutions of the different fluorene derivatives were prepared from corresponding stock ethanolic solutions (1.0 × 10^−4^ M). Excitation and fluorescence emission spectra were then recorded.

### 3.6. Fluorescence Studies of Fluorene Sensors Interaction with Native Insulin Protein

In order to test the sensing characteristics of fluorene derivatives for the detection of amyloid fibrils and aggregates, the influence of the presence of native insulin on the native fluorescence of fluorene derivatives was studied prior to this.

An aqueous solution of native insulin was prepared at a 1.0 × 10^−4^ M concentration, allowed to stand for 24 h, and then the exact concentration value was determined by UV–Vis absorption spectrophotometry (*ɛ* = 6191.5 M^−1^cm^−1^, λ_max_ = 276 nm). Solutions of fluorene derivatives and thioflavin T (ThT) were prepared at a concentration of 1.0 × 10^−6^ M in 50 μM phosphate buffer (PBS), pH 7.02. The exact concentration of ThT (*ɛ* = 36,000 M^−1^cm^−1^, λ_max_ = 412 nm) [54] and the concentration of fluorene derivatives was checked by UV–Vis absorption spectrophotometry using experimentally determined molar absorptivity. Titration of the fluorescence sensor was carried out with a native insulin solution 1.0 × 10^−4^ M concentration in 50 mM PBS buffer (pH = 7.02). Then, successive additions of 10 μL volumes of the protein solution were added to the buffered sensor solution. After each addition, fluorescence emission spectra were recorded using the wavelength of the excitation maximum.

### 3.7. Fluorene Sensors’ Interaction with Fibrils of β-amyloid Human Insulin Protein

Fresh solutions of fluorene derivatives, as well as ThT and GN8, were prepared in phosphate buffer (50 μM, pH = 7.02). Their concentrations were checked by UV–Vis absorption spectrophotometry and adjusted to the accurate value of 1.0 × 10^−6^ M. Subsequently, solutions of amyloid β-fibrils, freshly prepared according to methods A or B described in Section 3.4, were diluted to produce 1.0 × 10^−4^ M insulin fibrils in 50 mM PBS buffer, pH = 7.02). Aliquots of 10 μL of insulin fibril solutions, obtained using both methods A and B, were added to each of the sensor solutions (1.0 × 10^−6^ M). In these titrations, the sensor solutions were mixed with the amyloid insulin fibrils and maintained at 37 °C for 15 min in a water bath. After each addition and incubation, a stabilization period of 5 min at room temperature was allowed. Then, fluorescence emission spectra were recorded at the wavelengths corresponding to the excitation maxima for each compound. The increase in the fluorescence intensity of the band corresponding to the charge transfer process for the different fluorene derivatives allows for the calculation of the sensor fibril–amyloid affinity constants. The *K*_d_ values [57,63,64] were calculated from the experimental data (in duplicate sets) using GraphPad Prism 8 software (GraphPad Software Inc., La Jolla, CA, USA); the following equation, which relates to Michaelis–Menten kinetics, was employed for the study of binding saturation:

$$F=\frac{F_{max}\times \left[P\right]}{K_{d}+\left[P\right]}$$

*F*: fluorescence values (in arbitrary units, y-variable) obtained for the different concentration protein values; [*P*]: protein concentration (x-variable); *F_max_*: fluorescence intensity value at the *plateau* region; *K*_d_: dissociation constant.

### 3.8. Fluorescence Quenching for Evidencing Sensor–Protein Interaction

Titration of a 5.0 × 10^−6^ M insulin β-amyloid solution was carried out with increasing volumes of solutions of the fluorene derivatives **1**–**4**, as well as the reference compounds ThT and GN8. Protein solutions were prepared in phosphate buffer (50 mM PBS buffer, pH = 7.02). The insulin β-amyloid fibril solutions were incubated at 37 °C for 15 min after each of the sensor additions. They were then left at room temperature for 5 min, and their fluorescence emission spectra were recorded at the excitation fluorescence wavelength of 276 nm, corresponding to the excitation maximum of the protein. The final concentrations of the sensors varied from 0 to 1.25 × 10^−5^ M [55]. The existence of an inner filter effect was discarded because the fluorescence sensor solutions were below 0.05 absorbance units [65]. Considering the fluorescence quenching protein that occurs in the presence of fluorene derivatives and according to the following equation, the Stern–Volmer constant for this process can be determined.

$$\frac{F_{0}}{F}=1+K_{SV}\left[Q\right]$$

*F*_0_: fluorescence intensity in the absence of a quencher (fluorene derivatives); *F*: fluorescence intensity in the presence of varying concentrations of the quencher; *K_SV_*: Stern–Volmer constant; *Q*: concentration of the quencher (fluorene derivatives).

Thus, when plotting the values of the fluorescence intensity ratios versus the quencher concentration, a straight line should be obtained whose ordinate at the origin should be in the vicinity of 1.0. The value of the slope will correspond to the experimental value of the Stern–Volmer constant, which shows the effectiveness of the fluorescence quenching process and allows for the estimation of the magnitude of the sensor–protein interaction [66].

The affinity constants between fluorene-derived sensors and the amyloidogenic forms of insulin—the sensor–protein affinity constants—can be determined using the following equation:

$$log\frac{F_{0}-F}{F}=\log K+n\;\log\left[Q\right]$$

*F*_0_: fluorescence intensity in the absence of a quencher (fluorene derivatives); *F*: fluorescence intensity in the presence of different concentrations of the quencher; *K*: binding constant; *n*: number of binding sites; *Q*: concentration of the quencher (fluorene derivatives).

Plotting the logarithm of the quotient of the fluorescence intensities versus the logarithm of the quencher concentration should give a straight line whose ordinate is log *K*, and hence the affinity constant insulin protein fibrils–sensors can be deduced [54,63].

### 3.9. Determination of the Antioxidant Capacity of Fluorene Derivatives

#### 3.9.1. DPPH Method: 2,2-Diphenyl-1-picrylhydrazyl Radical Scavenging Activity

Stock solutions of each of the fluorene derivatives, as well as the model antioxidants, Trolox and curcumin, were prepared at a concentration of 1.0 × 10^−2^ M in pure ethanol or, if not completely dissolved, in an ethanol:DMSO mixture (90:10; *v*:*v*). Solutions of 300 μM of each sample compound were dissolved in a methanol:water (80:20; *v*:*v*) solution. Then, solutions of 9, 18, 30, 45, 60, 90, 120, and 300 μM concentrations of each fluorene derivative and the reference compounds were prepared in methanol:water. To 100 μL methanol:water (80:20; *v*:*v*) solution of each compound at the above mentioned concentrations, 200 μL of 100 μM methanolic DPPH^•^ were added and placed in the 96-well microliter plate. The plate was kept in the dark for 30 min. After that, the absorbance was measured by a microplate reader at 517 nm in order to quantify the decrease in the absorbance. All samples and reference compounds at the different concentrations were prepared and measured in triplicate sets. Blanks were prepared to be taken into account in the total antioxidant capacity calculations. A first blank (B1) was prepared in methanol:water (80:20; *v*:*v*) with the suitable proportion of DMSO (final volume 300 μL). A second blank (B2) was then prepared with 100 μL of methanol:water (80:20; *v*:*v*) and 200 μL of DPPH 100 μM. The blanks were measured in triplicate wells and used to calculate the amount of remaining DPPH^•^. The absorbance at 517 nm decreased proportionally to the increases of non-radical forms of DPPH and, therefore, to the presence of increasing concentrations of compounds with antioxidant activity.

$$\Delta A=A_{Control}-A_{Sample}$$

*A_Control_* is the absorbance value corresponding to the solution blank B2 (higher absorbance), DPPH 100 μM; *A_Sample_* is the absorbance value corresponding to the different concentration values of each antioxidant assayed.

The absorbance increase values (Δ*A*) were plotted against the antioxidant concentration values and the values of antioxidant capacity (DPPH) were deduced from the slopes obtained in the range corresponding to the straight line, and then the total antioxidant capacity was quantified by comparison with Trolox. A value of 1 was assigned to the Trolox slope value in the same experimental conditions [67,68,69,70].

#### 3.9.2. FRAP Method: Ferric Ion Reducing Antioxidant Power

A commercial FRAP kit purchased from Merck (Madrid, Spain) was used for this assay. Stock solutions of each of the fluorene derivatives, as well as the model antioxidants, Trolox and curcumin, were prepared at a concentration of 1.0 × 10^−2^ M in ethanol or acetone. From these stock solutions, fresh ethanolic solutions of 600 μM concentration were prepared for the fluorene derivatives to be studied, alongside solutions of Trolox and curcumin prepared in the same way, which were used as references.

All the solutions of the compounds to be tested, as well as the kit solutions, i.e., the standard Fe^2+^ solution, the FeCl_3_, the buffer, and the probe solutions, were kept in an ice bath throughout the preparation of the 96-microwell plate. Furthermore, the plate was also maintained in an ice bath during the plate charge. The Fe^2+^ calibration curve was prepared by taking 0, 2, 4, 6, 8, and 10 μL of the 2 mM ferrous standard solution and making up to 10 μL with the FRAP assay buffer. The 10 μL of each of these solutions was placed in the wells and 190 μL of the reaction mixture containing the FRAP probe was added to each well. All points of the calibration curve were performed in duplicate, as each plate has its own Fe^2+^ calibration curve. The final Fe^2+^ concentrations in the wells were 0, 20, 40, 60, 80, and 100 μM.

A total of 10 μL of the ethanolic solutions with a 600 μM concentration of the fluorene derivatives and the references, Trolox and curcumin, were deposited in the corresponding wells, and then 190 μL of the reaction mixture with the FRAP probe was added to a final concentration of samples and references in the wells of 30 μM. In addition, a positive control was prepared with 4 μL of FRAP-positive control, 6 μL of buffer, and 190 μL of reaction mixture with the FRAP probe. Negative controls were also prepared for each of the compounds to be tested by adding 10 μL of compound or reference compound of 600 μM concentration and 190 μL of reaction mixture without the FRAP probe, while an instrumental blank was prepared by mixing 10 μL of ethanol with 190 μL of reaction mixture without the FRAP probe. All compounds, references, blank, positive, and negative controls were assayed in triplicate.

Then, the plate containing the Fe^2+^ standard solutions as well as the test compounds, blanks, and controls, was incubated in the dark at 37 °C for 1 h. Subsequently, the absorbance values were determined at 594 nm in the spectrophotometer with a multi-well plate reader. The absorbance values of the sample antioxidant compounds were corrected by subtracting the corresponding absorbance values of the blanks, and then they were introduced in the calibration curve to calculate the ferrous equivalents. The TEAC (total equivalent antioxidant capacity) was set by arbitrarily assigning a value of 1.00 to the number of nanomoles of Fe^2+^ corresponding to Trolox 30 μM (in the plate) and setting those of the fluorene derivatives and curcumin by comparing against this value [65,71].

## 4. Conclusions

The preservation of insulin injections is complicated by the formation of amyloid insulin conformations, which have a negative influence on the bioavailability of the injected protein due to their lower solubility and, moreover, exert undesired effects upon administration. The detection of insulin amyloid fibrils is thus of great relevance from the point of view of pharmaceutical technology.

We investigate here the usefulness of rigid analogues of GN8, a cellular prion protein ligand, for the detection of insulin amyloid fibrils. These compounds, derived from a fluorene framework, undergo an enhancement in the fluorescence emission spectra in the presence of insulin amyloid aggregates via intramolecular charge transfer between the substituents and the fluorene core, showing that they can be used as fluorescence probes for detecting abnormal conformations of proteins with protein–sensor dissociation constants in the range of 5–10 μM. Moreover, the observed overlap of the emission spectra of protein and excitation spectra of the fluorene derivatives, which is particularly efficient in the case of compound **4**, allows for an intermolecular resonance energy transfer effect (RET) that leads to a quenching effect of the protein fluorescence by the sensors that can be exploited for the sensitive detection of abnormal insulin conformations. The dissociation constant, which is similar to the values obtained with GN8 and the thioflavin T gold standard, and the Stern–Volmer quenching constant for compound **4** provide quantitative evidence of the formation of the protein–sensor complex. As protein misfolding diseases are closely associated with oxidative stress, the antioxidant capacity of the fluorene derivatives was evaluated, which showed good antioxidant behavior in DPPH assays.

## Figures and Tables

**Table 1:** Dissociation constants (*K*_d_) obtained for the fluorene sensors and fibrils of insulin-derived amyloid protein. The correlation obtained (R^2^) was higher than 0.98 in all cases.

Compound ^1^	*K*_d_ ^2^
Thioflavin T	16.46
**1**	5.59
**2**	5.60
**4**	10.12

^1^ No adjustment to the model was obtained for compound **3**. ^2^ *K*_d_: dissociation constant (μM).

**Table 2:** Fluorescence quenching studies. Stern–Volmer (*K*_SV_) and binding constants (*K*) obtained for the fluorene derivatives and the reference compounds with fibrils of insulin β-amyloid protein. The correlation obtained (R^2^) was higher than 0.98 in all cases.

Compound	*K*_SV_	*K*
Thioflavin T	2.11 × 10^4^	1.26 × 10^3^
GN8	1.63 × 10^4^	1.01 × 10^4^
**4**	1.09 × 10^5^	1.46 × 10^5^

## Data Availability

Data are contained within the article and Supplementary Materials.

## Supplementary Materials

The following supporting information can be downloaded at: https://www.mdpi.com/article/10.3390/molecules29061196/s1, Copies of NMR spectra. Figure S1: UV–Vis absorption spectra of fluorene sensors in ethanolic solution; Figure S2: Effect of the additions of increasing volumes of native insulin on the fluorescence emission spectra of reference compounds; Figure S3: Effect of the additions of increasing volumes of insulin fibrils/aggregates on the fluorescence emission spectra of reference compound thioflavin T; Figure S4: Comparison of the increase in the fluorescence intensity of the reference compound thioflavin T employing method A and method B for inducing insulin β-amyloid protein aggregation. Figure S5: Fluorescence emission spectra of compound **1** and fixed concentration of amyloid fibrils of insulin. Figure S6: Fluorescence emission spectra of fluorene (5 × 10^−6^ M) in the presence of increasing concentrations of amyloid fibrils of insulin. Figure S7: Fluorescence emission spectra (λ_ex_ = 275 nm) of amyloid insulin in the presence of increasing concentrations thioflavin T. Figure S8: Calculation of the binding constant for the quenching by thioflavin T of the amyloid insulin fluorescent emission at 305 nm. Figure S9: Calculation of the Stern–Volmer constant (*K*_SV_) for the quenching by thioflavin T of the amyloid insulin fluorescent emission at 305 nm. Figure S10: Fluorescence of amyloid insulin quenched by GN8. Figure S11: Calculation of the binding constant for the quenching by GN8 of the amyloid insulin fluorescent emission at 305 nm. Figure S12: Calculation of the Stern–Volmer constant (*K*_SV_) for the quenching by GN8 of the amyloid insulin fluorescent emission at 305 nm.
