# Supplementary material for: Fluorimetric Detection of Insulin Misfolding by Probes Derived from Functionalized Fluorene Frameworks
Source: Molecules. 2024 Mar 7;29(6):1196. doi: 10.3390/molecules29061196 (PMC10975426; doi:10.3390/molecules29061196)
Supplement: Supplementary file 1 [file molecules-29-01196-s001.zip › molecules-2877987-supplementary.pdf]

Supporting Information

Fluorimetric detection of insulin misfolding by probes derived from functionalized fluorene frameworks

Álvaro Sarabia-Vallejo [1], Ana Molina [2], Mónica Martínez-Orts [2], Alice D'Onofrio [1], Matteo Staderini [1], Maria Laura Bolognesi [3], M. Antonia Martín [2,*], Ana I. Olives [2], and J. Carlos Menéndez [1,*]

[1] Unidad de Química Orgánica y Farmacéutica, Departamento de Química en Ciencias Farmacéuticas, Facultad de Farmacia, Universidad Complutense, 28040 Madrid, Spain; josecm@ucm.es

[2] Unidad de Química Analítica, Departamento de Química en Ciencias Farmacéuticas, Facultad de Farmacia, Universidad Complutense, 28040 Madrid, Spain; mantonia@farm.ucm.es

[3] Department of Pharmacy and Biotechnology, Alma Mater Studiorum - University of Bologna, Via Belmeloro 6, 40126, Bologna, Italy.

Table of contents

1. Copies of NMR spectra S2
2. UV-Vis and fluorescence experiments: Figures S1 – S12 S9
3. Tables S19

S1. Copies of NMR spectra

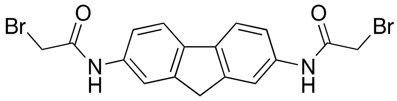

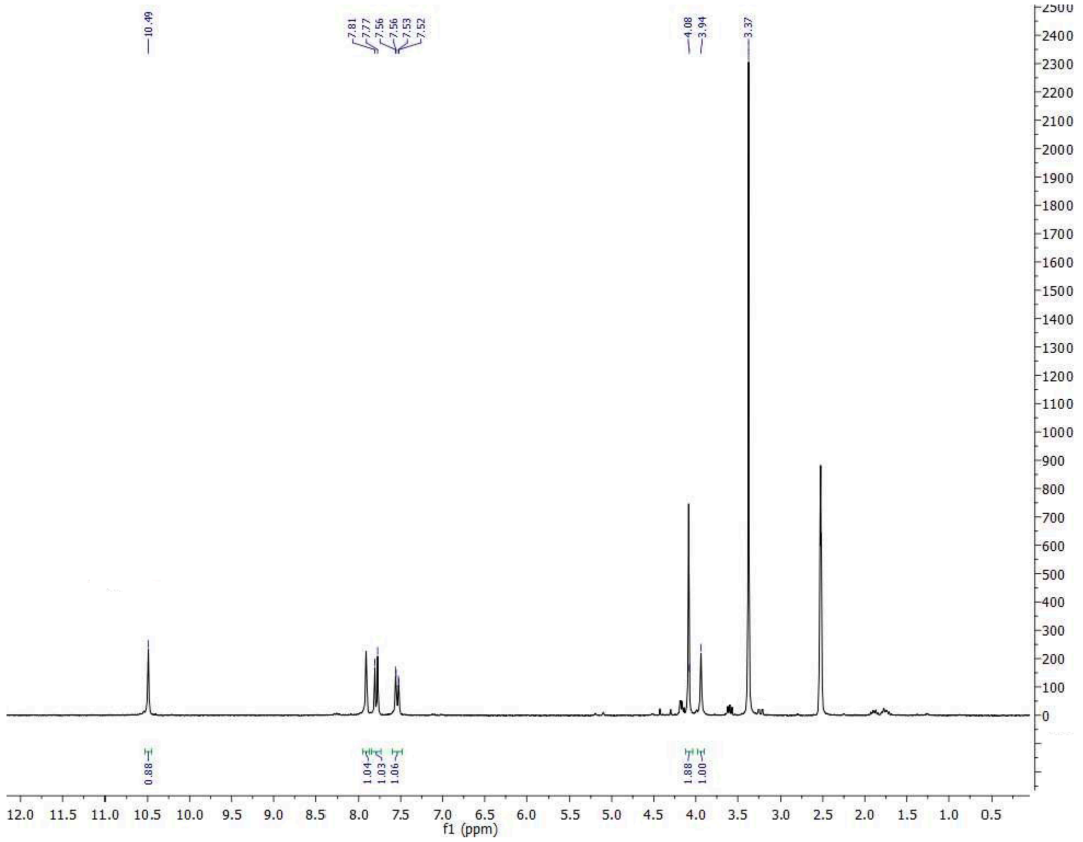

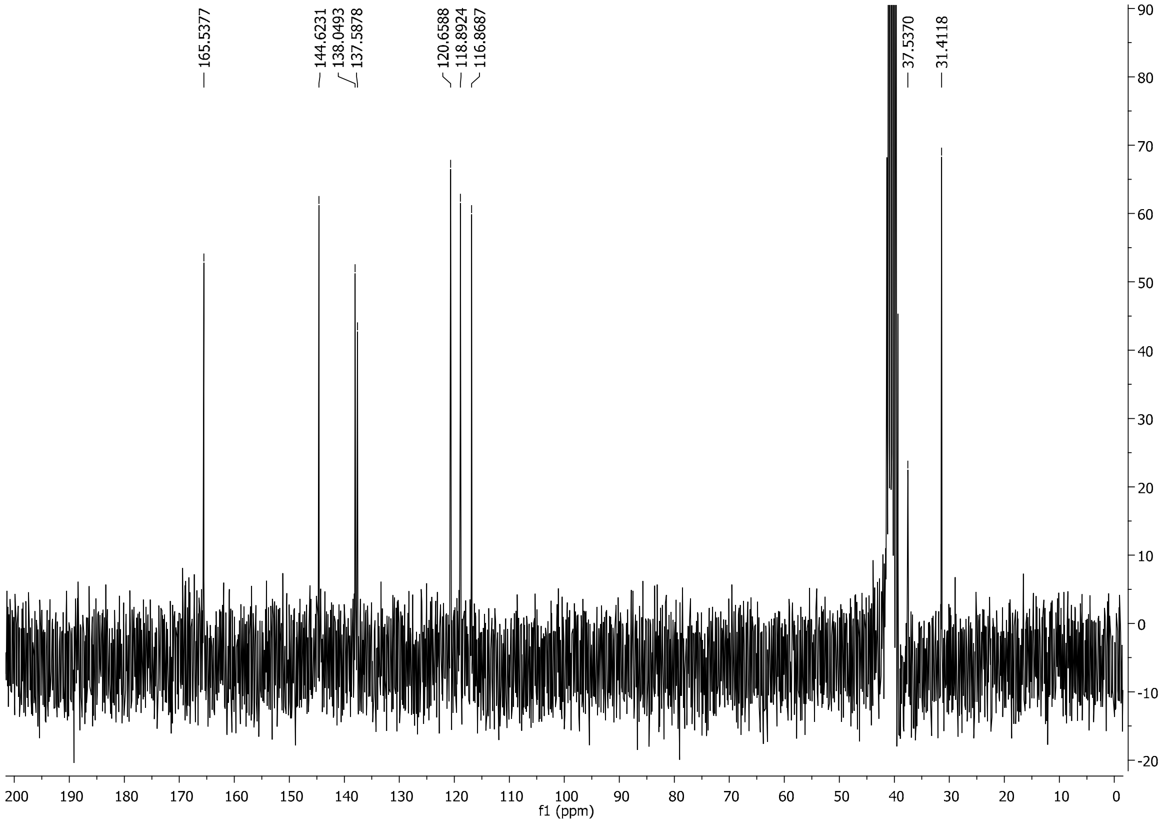

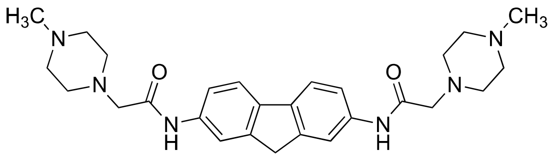

H3C
N
N
O
N
H
CH3

1

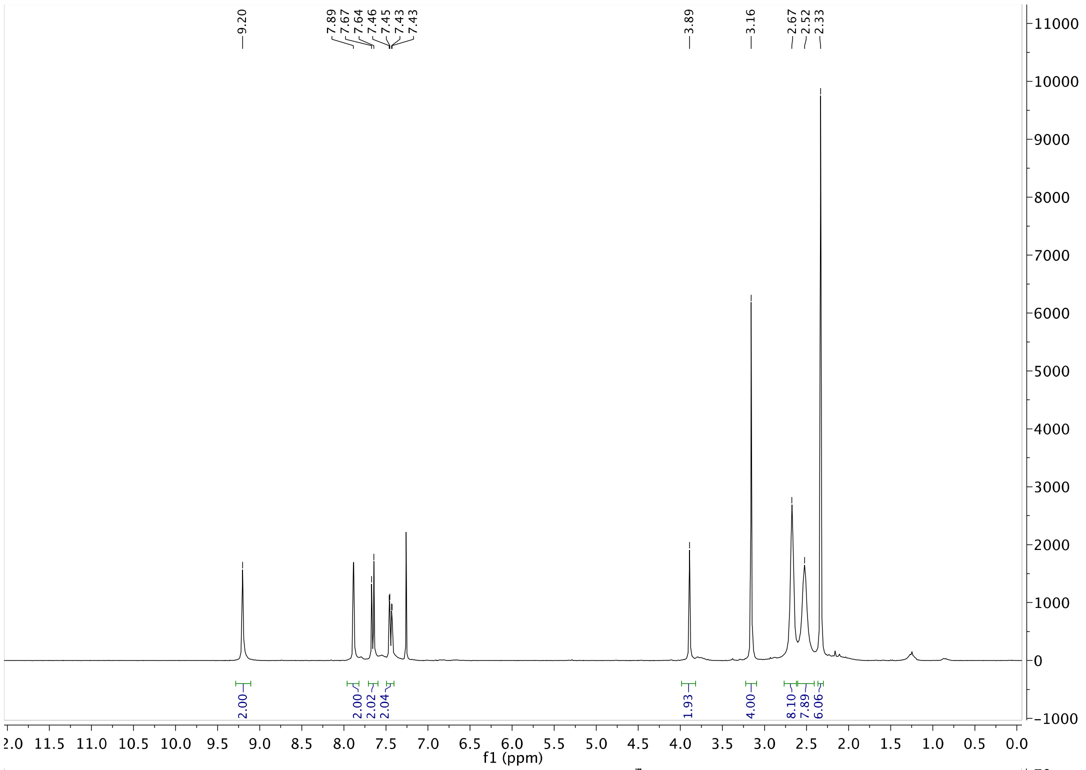

9.20
7.89
7.67
7.64
7.46
7.45
7.43
7.43
3.89
3.16
2.67
2.52
2.33
2.00
2.00
2.02
2.04
1.93
4.00
8.10
7.89
6.06
f1 (ppm)

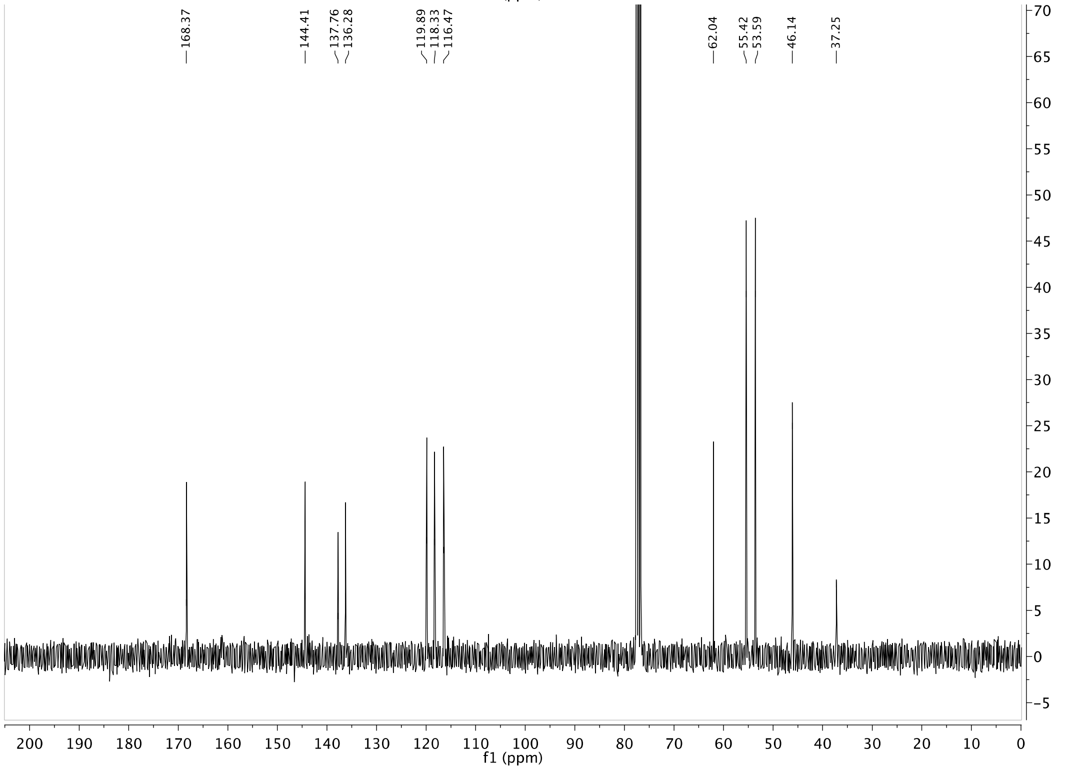

168.37
144.41
137.76
136.28
119.89
118.33
116.47
62.04
55.42
53.59
46.14
37.25
f1 (ppm)

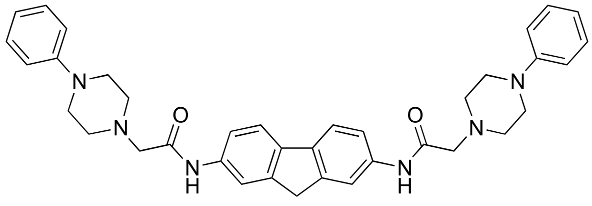

2

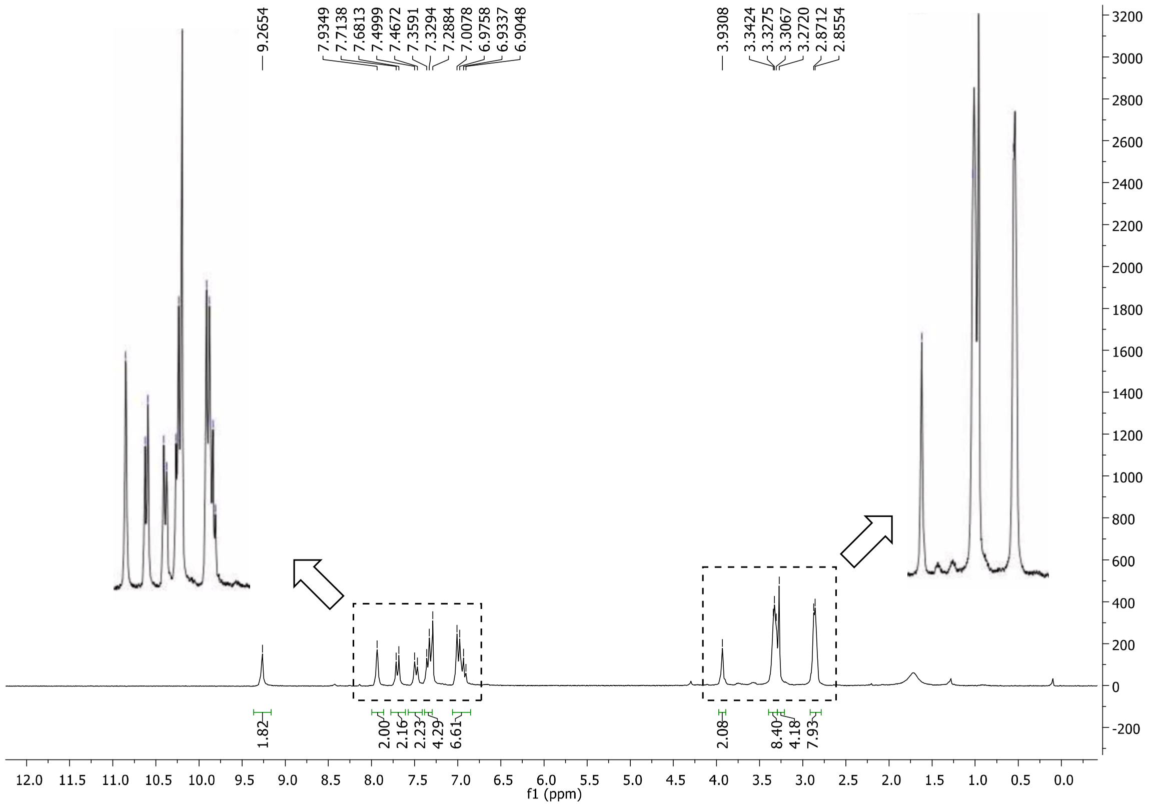
9.2654
7.9349
7.7138
7.6813
7.4999
7.4672
7.3591
7.3294
7.2884
7.0078
6.9758
6.9337
6.9048
3.9308
3.3424
3.3275
3.3067
3.2720
2.8712
2.8554
1.82
2.00
2.16
2.23
4.29
6.61
2.08
8.40
4.18
7.93
f1 (ppm)

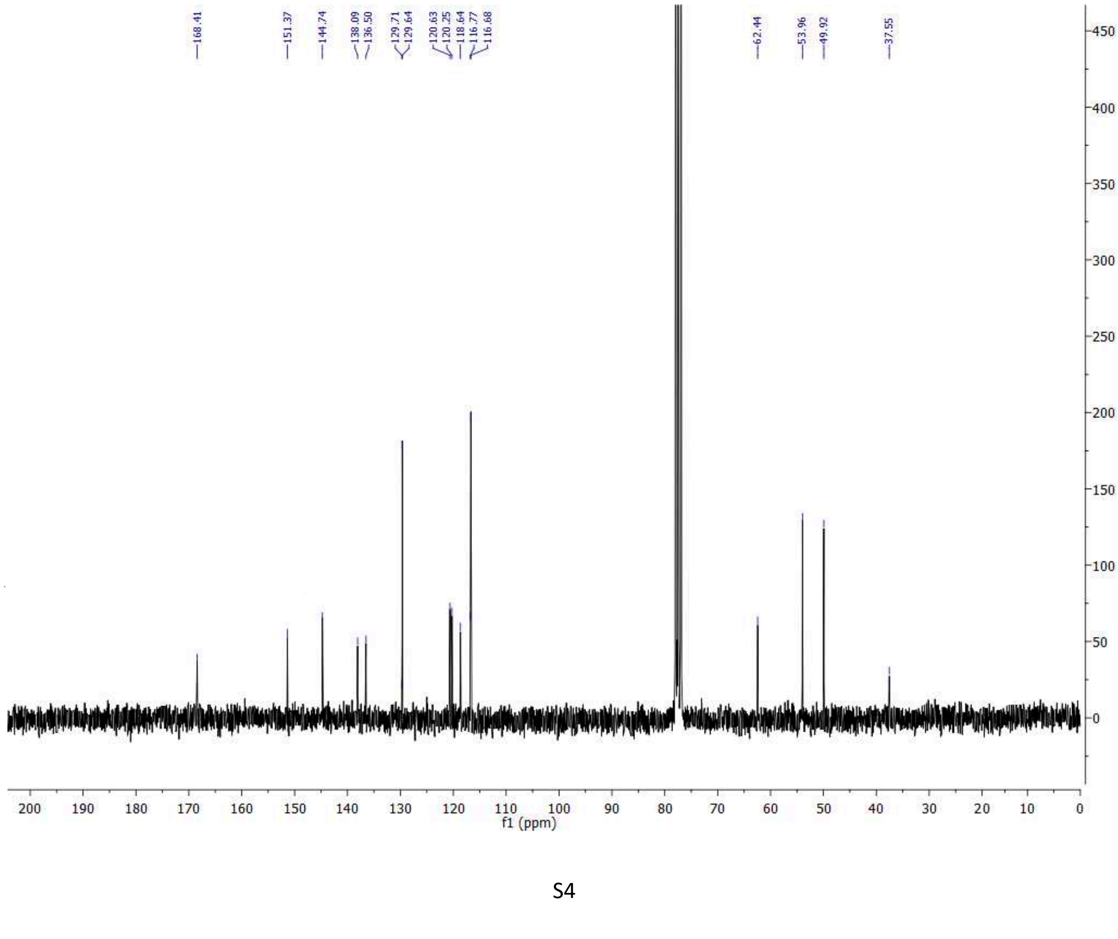
168.41
151.37
144.74
138.09
136.50
129.71
129.64
120.63
120.25
118.64
116.77
116.68
62.44
53.96
49.92
37.55
f1 (ppm)

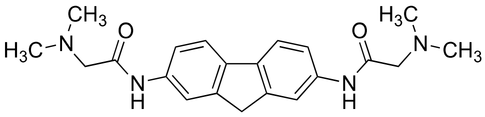

CH3
H3C
N
O
NH
H3C
N
CH3
O
NH

3

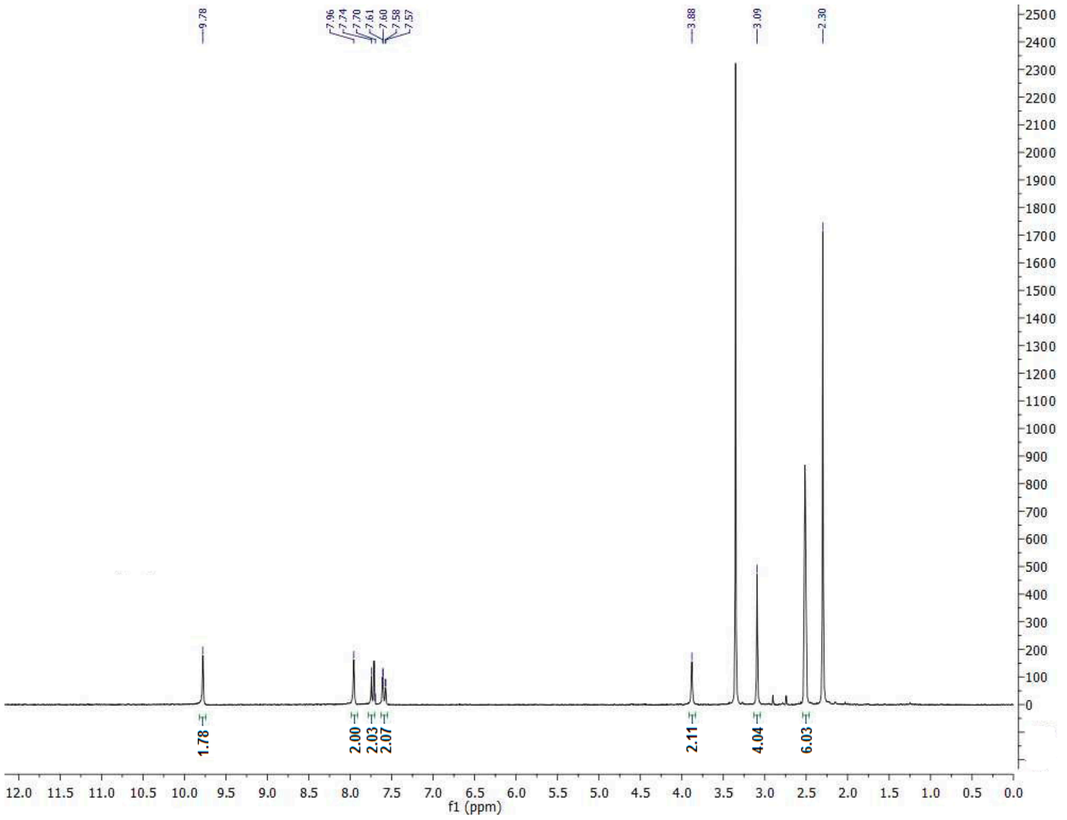

9.78
7.96
7.74
7.70
7.61
7.60
7.58
7.57
3.88
3.09
2.30
1.78
2.00
2.03
2.07
2.11
4.04
6.03
f1 (ppm)
12.0 11.5 11.0 10.5 10.0 9.5 9.0 8.5 8.0 7.5 7.0 6.5 6.0 5.5 5.0 4.5 4.0 3.5 3.0 2.5 2.0 1.5 1.0 0.5 0.0
2500 2400 2300 2200 2100 2000 1900 1800 1700 1600 1500 1400 1300 1200 1100 1000 900 800 700 600 500 400 300 200 100 0

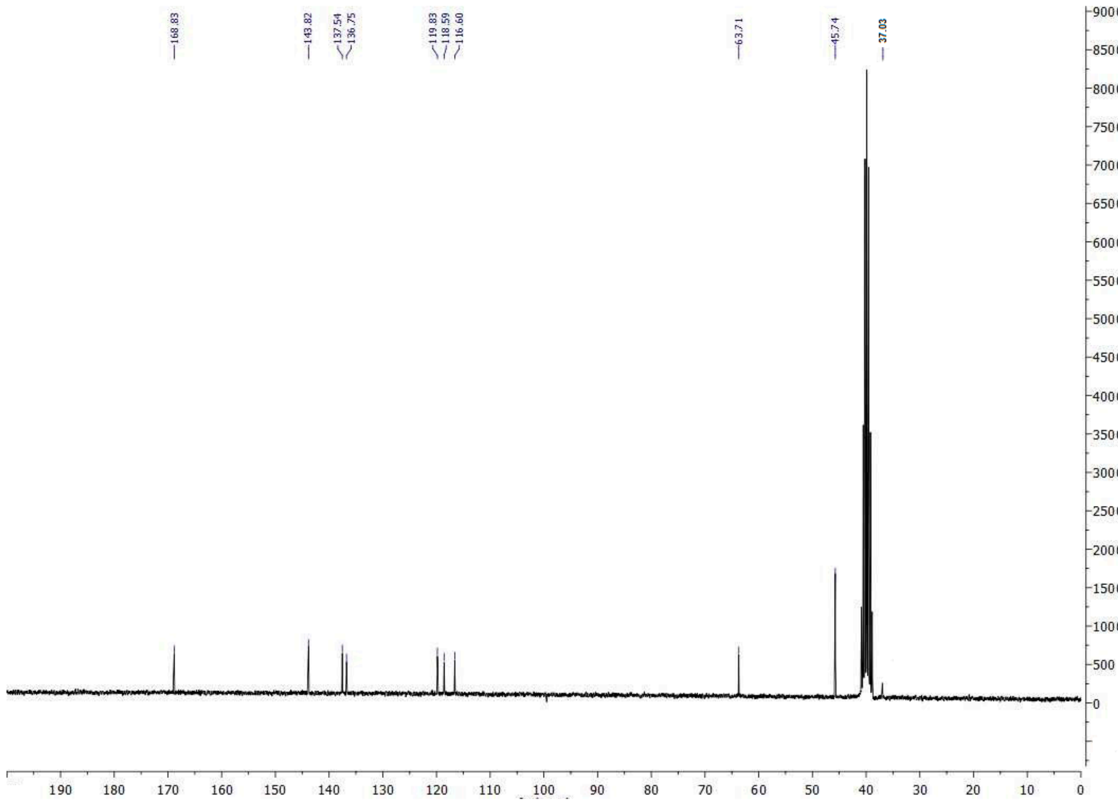

168.83
143.82
137.54
136.75
119.83
118.59
116.60
63.71
45.74
37.03
190 180 170 160 150 140 130 120 110 100 90 80 70 60 50 40 30 20 10 0
9000 8500 8000 7500 7000 6500 6000 5500 5000 4500 4000 3500 3000 2500 2000 1500 1000 500 0

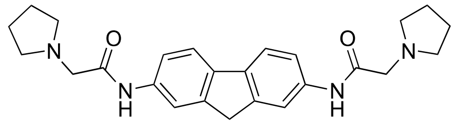
O
N
H
O
N
H
N
N

4

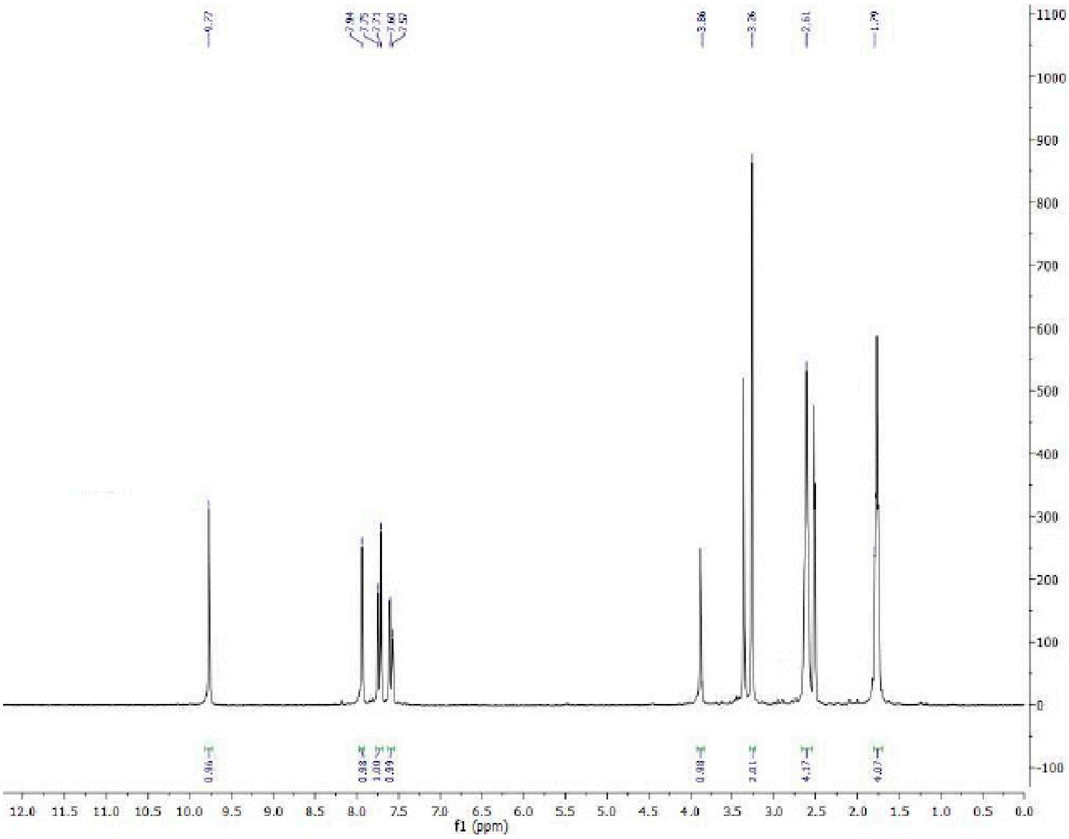
9.77
7.94
7.75
7.73
7.60
7.57
3.86
3.26
2.61
1.79
0.96
0.98
1.00
0.99
0.98
2.01
4.17
4.07
f1 (ppm)

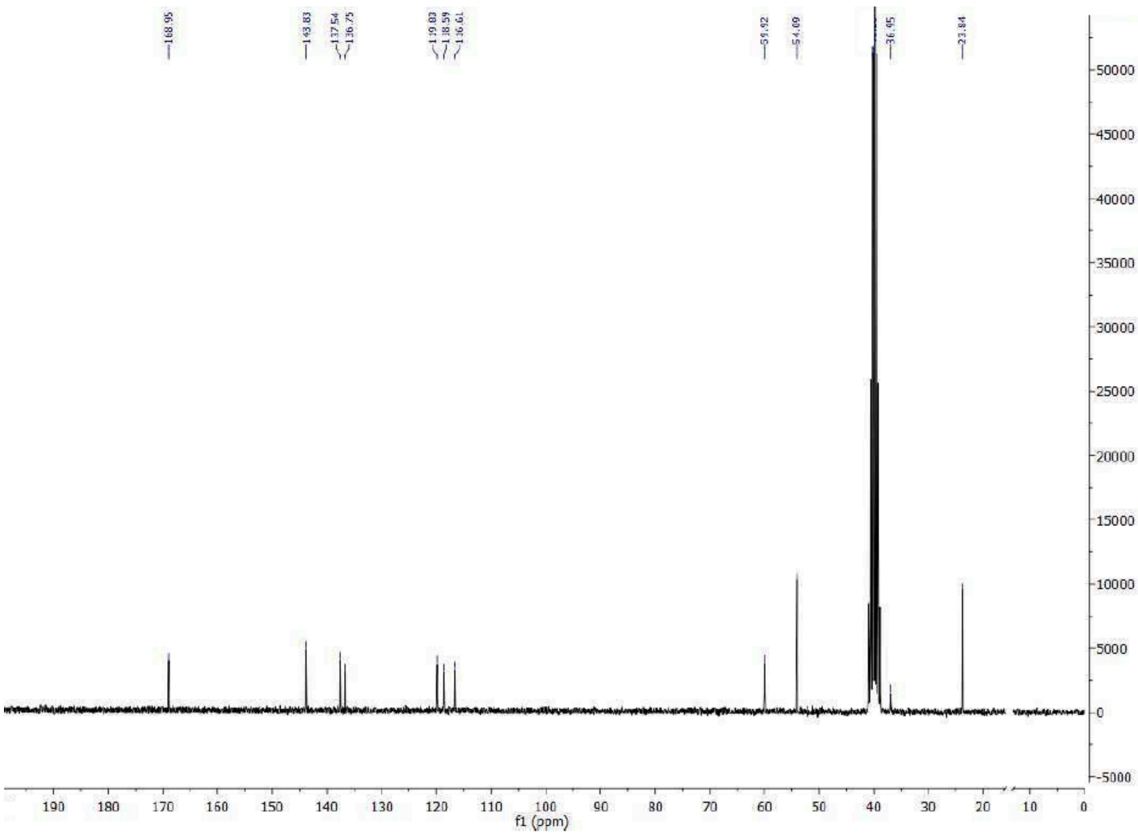
168.95
143.83
137.54
136.75
119.83
118.59
116.61
59.92
54.00
36.95
23.84
f1 (ppm)

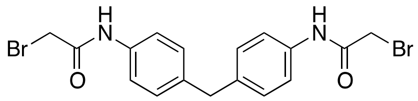
Br
H
N
O
H
N
O
Br

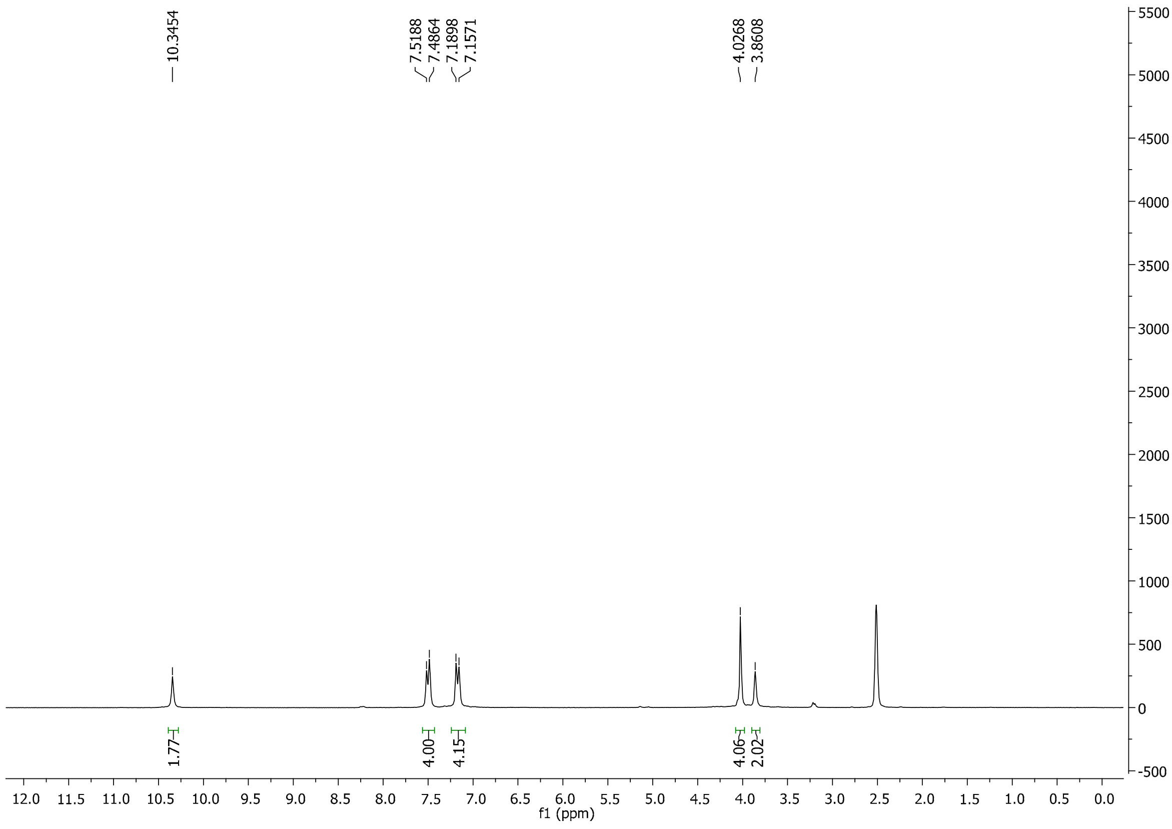
10.3454
7.5188
7.4864
7.1898
7.1571
4.0268
3.8608
1.77
4.00
4.15
4.06
2.02
12.0 11.5 11.0 10.5 10.0 9.5 9.0 8.5 8.0 7.5 7.0 6.5 6.0 5.5 5.0 4.5 4.0 3.5 3.0 2.5 2.0 1.5 1.0 0.5 0.0
f1 (ppm)
5500
5000
4500
4000
3500
3000
2500
2000
1500
1000
500
0
-500

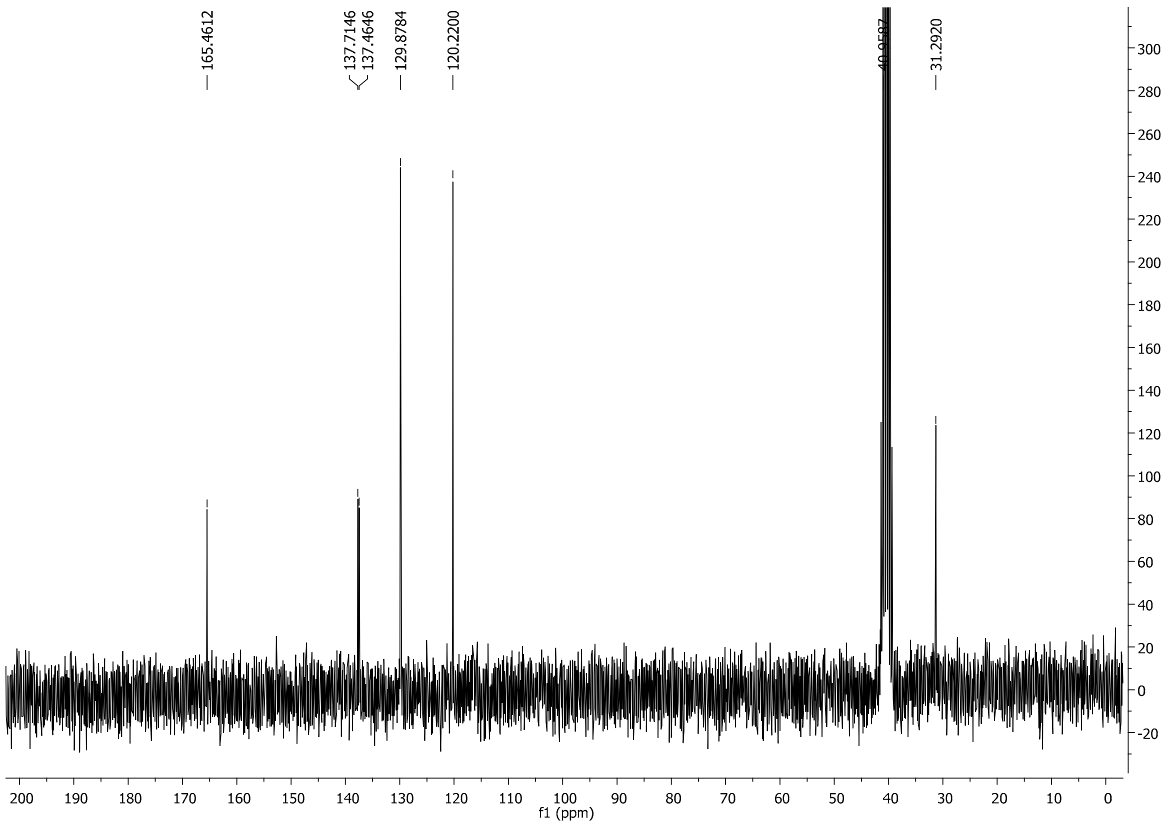
165.4612
137.7146
137.4646
129.8784
120.2200
31.2920
200 190 180 170 160 150 140 130 120 110 100 90 80 70 60 50 40 30 20 10 0
f1 (ppm)
300
280
260
240
220
200
180
160
140
120
100
80
60
40
20
0
-20

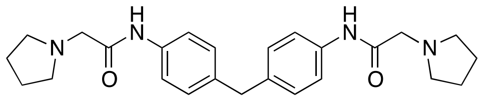

GN8

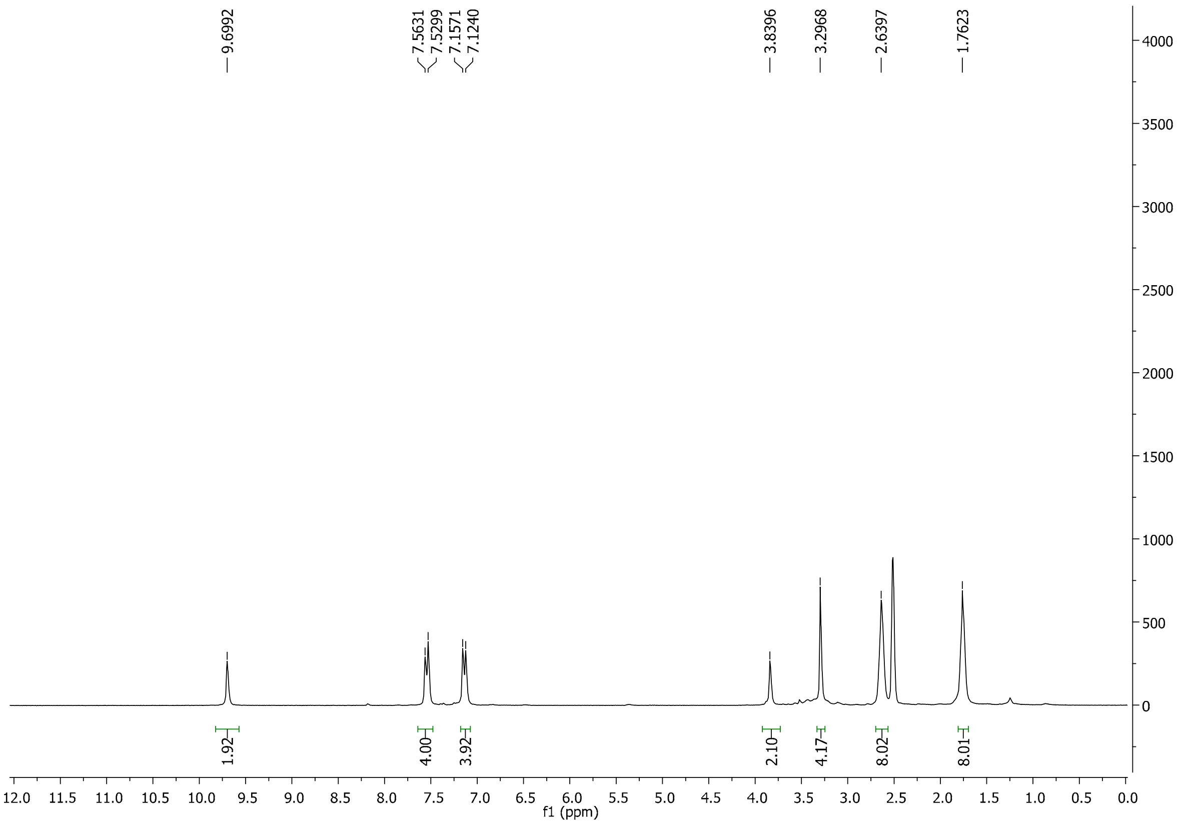
9.6992
7.5631
7.5299
7.1571
7.1240
3.8396
3.2968
2.6397
1.7623
1.92
4.00
3.92
2.10
4.17
8.02
8.01
f1 (ppm)

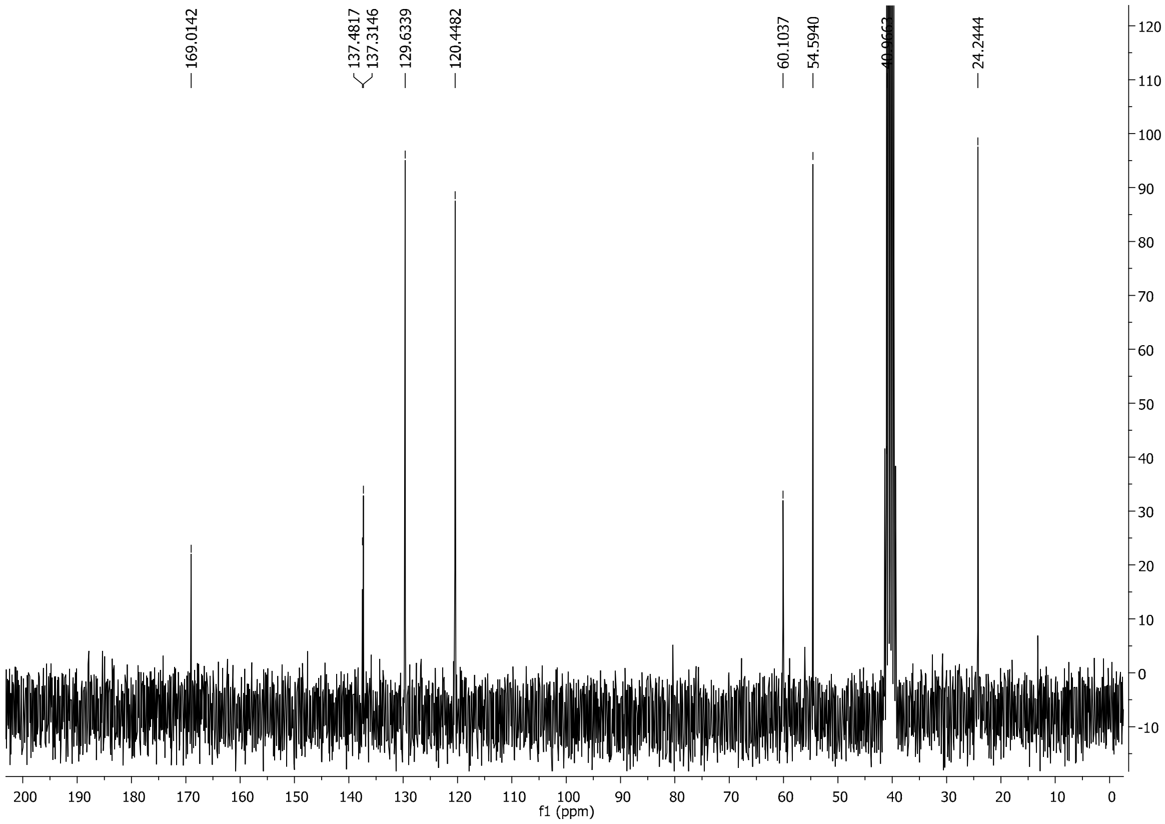
169.0142
137.4817
137.3146
129.6339
120.4482
60.1037
54.5940
24.2444
f1 (ppm)

S2. UV-Vis and fluorescence experiments

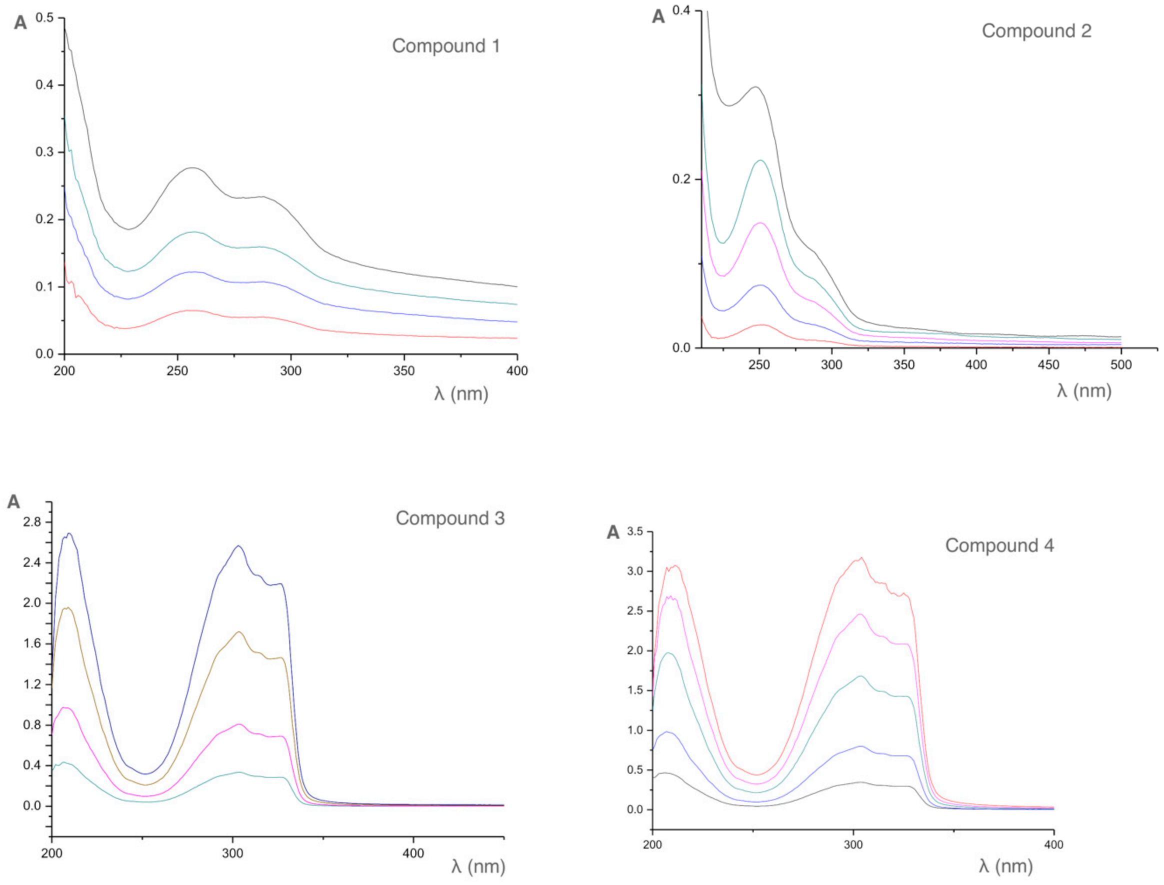

Figure S1: UV-Vis absorption spectra of fluorene sensors in ethanolic solution. Range of concentrations 1.0 x 10^{-5} M - 1.0 x 10^{-4} M.

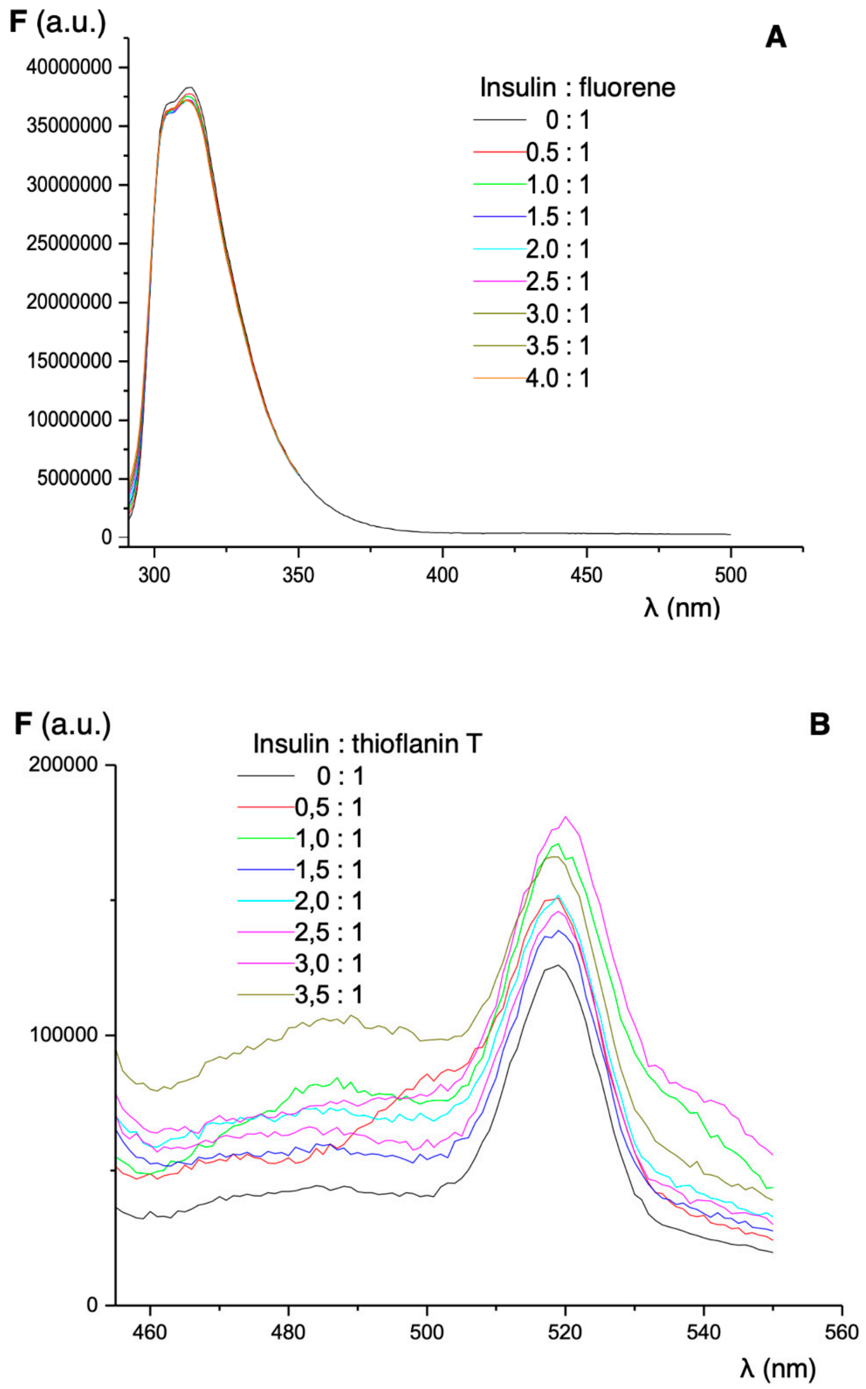

Figure S2: Effect of the additions of increasing volumes of native insulin on the fluorescence emission spectra of reference compounds. A. fluorene at λ_{ex} = 266 nm and B. Thioflavin T at λ_{ex} = 440 nm. Protein:sensor molar ratios are in the inset. The peak at 520 nm corresponds to Raman dispersion.

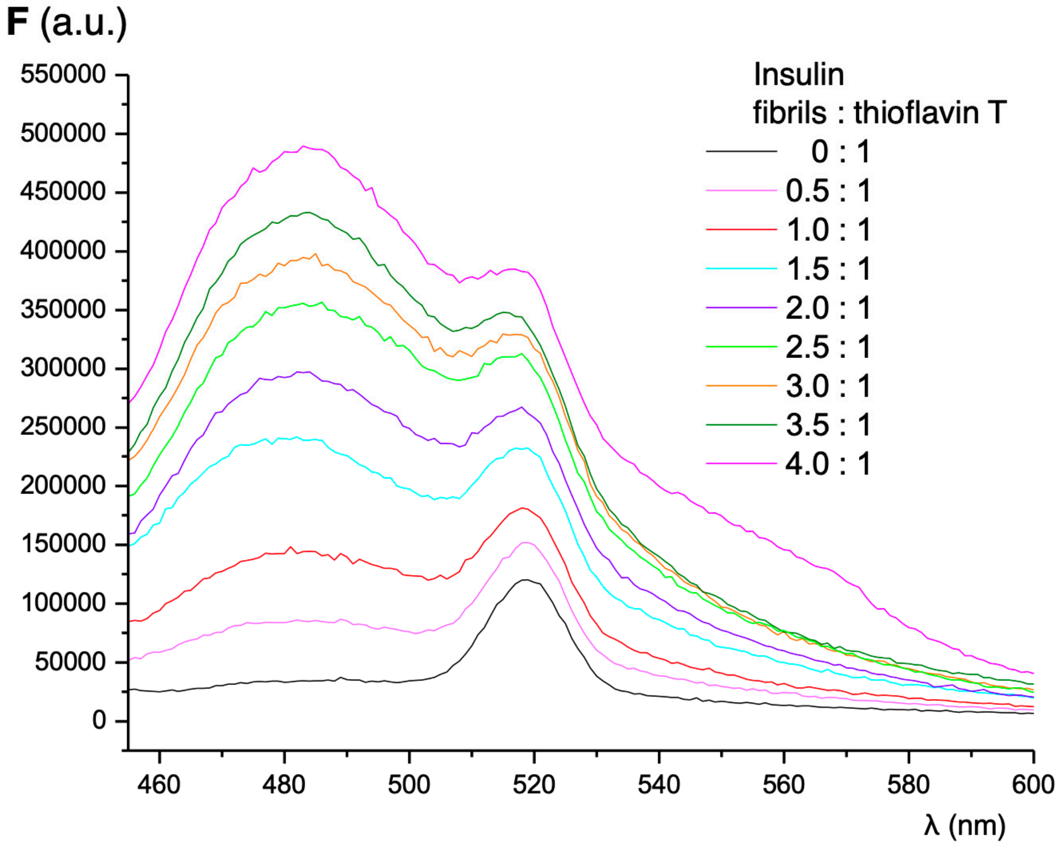

Figure S3: Effect of the additions of increasing volumes of insulin fibrils/aggregates on the fluorescence emission spectra of reference compound thioflavin T at λ_{ex} = 440 nm employing method A for protein aggregation. Protein:sensor molar ratios are in the inset. The peak at 520 nm corresponds to Raman dispersion.

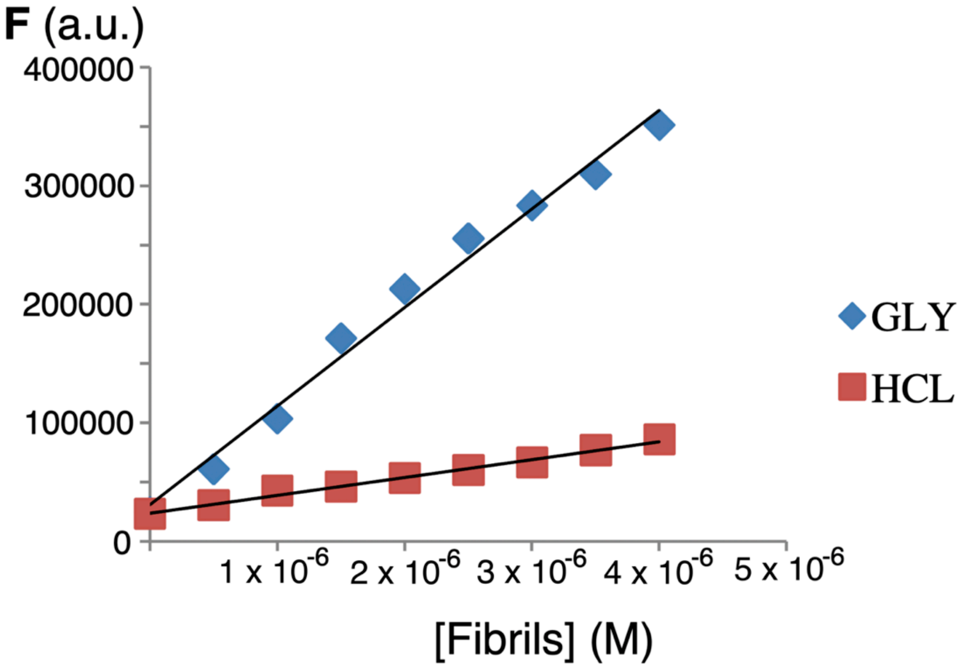

Figure S4: Comparison of the increase in the fluorescence intensity of the reference compound thioflavin T (λ_{ex} = 440; λ_{em} = 490 nm) employing method A (GLY) and method B (HCL) for inducing insulin β-amyloid protein aggregation.

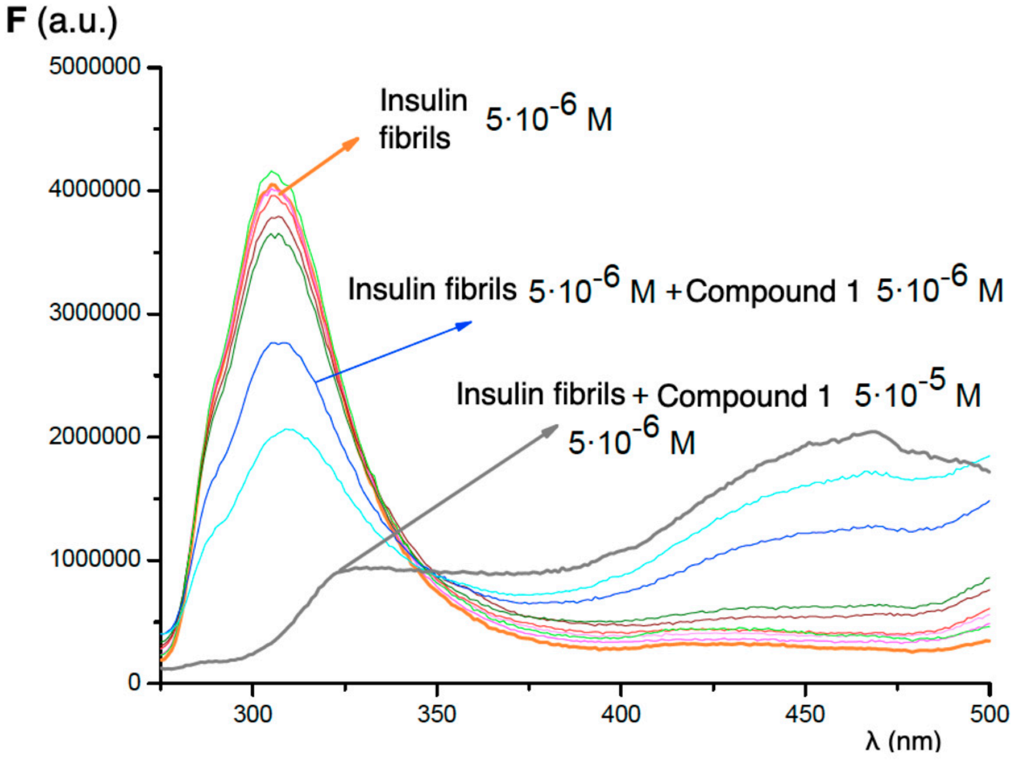

Figure S5: Fluorescence emission spectra of compound **1** (concentration varies from 0 to 5 x 10^{-5} M) at λ_{ex} = 260 nm and fixed concentration of amyloid fibrils of insulin (5 x 10^{-6} M).

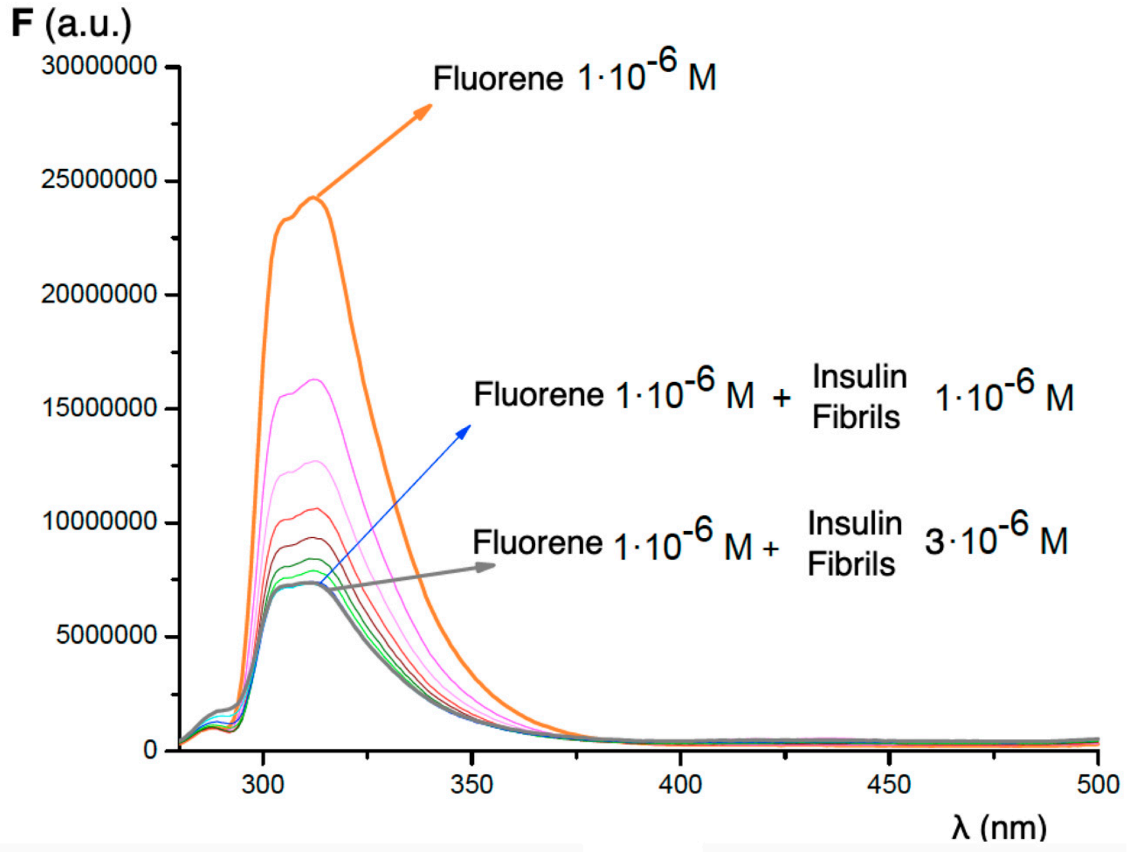

Figure S6: Fluorescence emission spectra of fluorene (5 x 10^{-6} M) in the presence of increasing concentrations of amyloid fibrils of insulin (concentration varies from 0 to 3 x 10^{-6} M) at λ_{ex} = 261 nm.

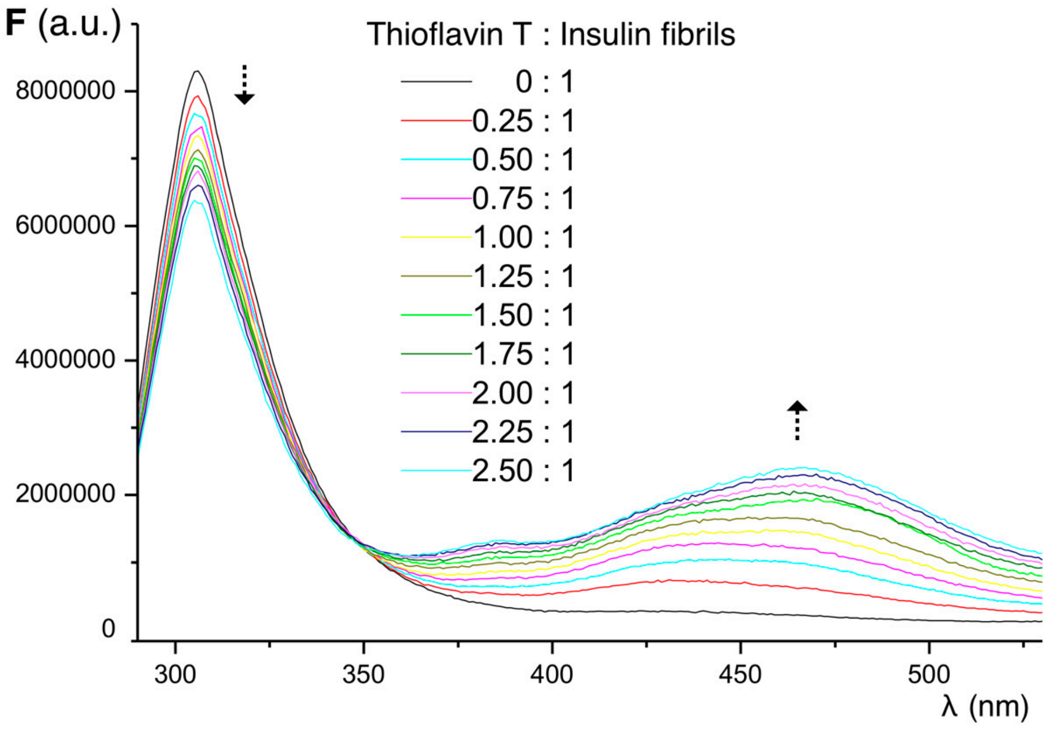

Figure S7: Fluorescence of amyloid insulin quenched by thioflavin T. Amyloid insulin fibrils obtained according to method A. Concentration of insulin fibrils 5 x 10^{-6} M. The concentration of thioflavin varies from 0 to 12.5 x 10^{-6} M; at λ_{ex} = 275 nm.

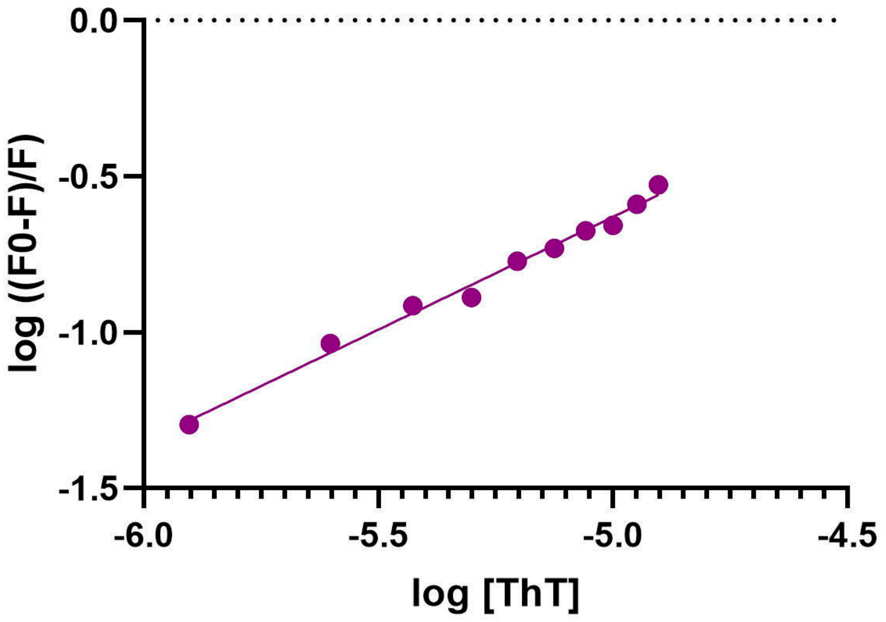

Figure S8. Calculation of the binding constant for the quenching by thioflavin T of the amyloid insulin fluorescent emission at 305 nm

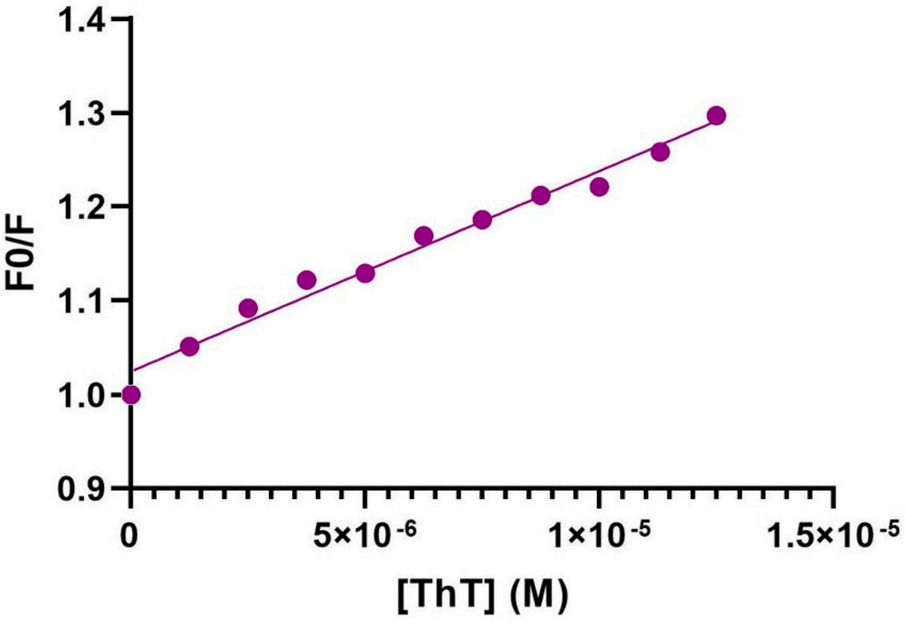

Figure S9. Calculation of the Stern-Volmer constant (K_{SV}) for the quenching by thioflavin T of the amyloid insulin fluorescent emission at 305 nm

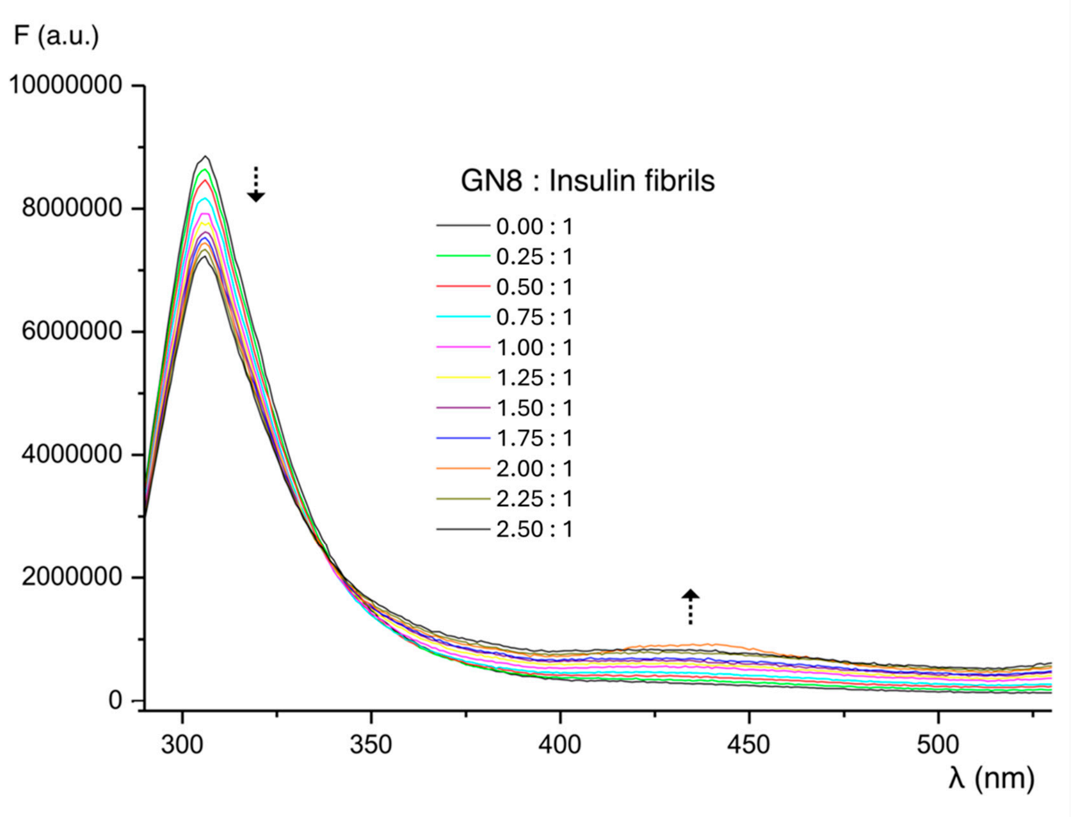

Figure S10: Fluorescence of amyloid insulin quenched by GN8. Amyloid insulin fibrils obtained according to method A. Concentration of insulin fibrils 5 x 10^{-6} M. Concentration of GN8 varies from 0 to 12.5 x 10^{-6} M; at λ_{ex} = 275 nm.

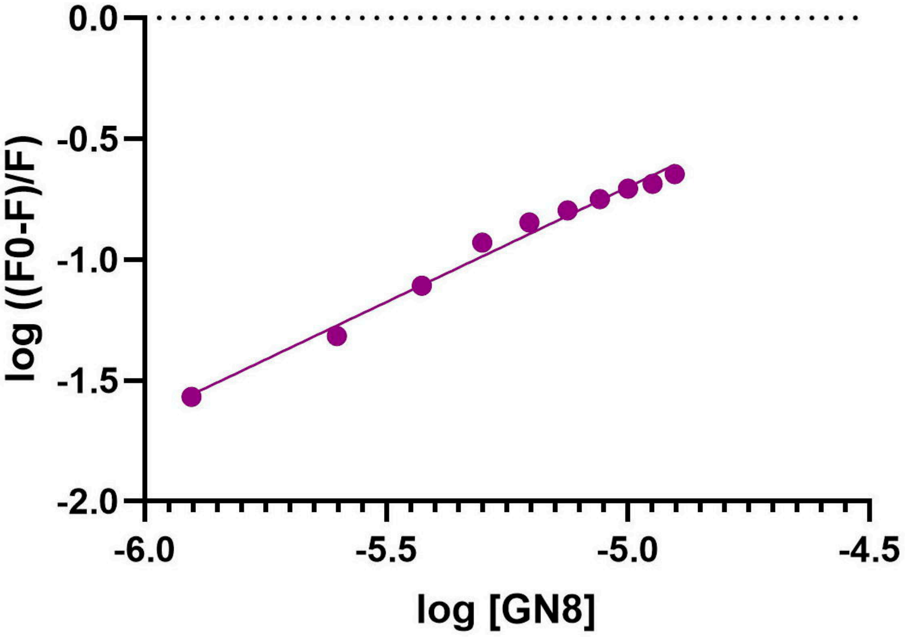

Figure S11. Calculation of the binding constant for the quenching by GN8 of the amyloid insulin fluorescent emission at 305 nm

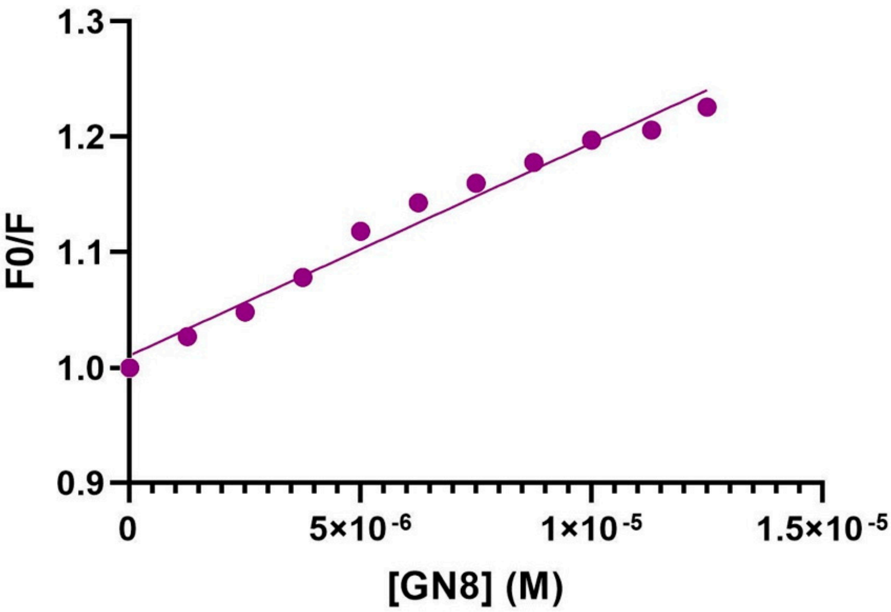

Figure S12. Calculation of the Stern-Volmer constant (K_{SV}) for the quenching by GN8 of the amyloid insulin fluorescent emission at 305 nm

S3. Tables

Table S1: Molar absorptivities at the maximum absorption wavelengths obtained in pure ethanol for the fluorene derivatives studied

Compound	***λ_{max} (nm) and molar absorptivity (log ε)***
**1**	256 (3.446), 289 (3.368)
**2**	250 (3.469), 290 (2.884)
**3**	304 (4.520), 327 (4.454)
**4**	304 (4.503), 326 (4.432)

Table S2: UV-Visible absorption maxima of the fluorene derivatives studied in different solvents. Fluorene derivatives concentration 2.0 x 10^{-5} M

	**UV-Visible absorption maxima (nm)**			
Compound	***Cyclohexene***	***Ethanol***	***Acetonitrile***	***Water***
**1**	260, 287	256, 289	257, 287	254, 287
**2**	252, 287 (sh)	250, 290	248, 290 (sh)	257, 289 (sh)
**3**	303, 315	304, 327	305, 326	296, 311
**4**	305, 329	304, 326	305, 326	296, 311

sh: shoulder

Table S3: Fluorescence emission maxima (excitation wavelength in parenthesis) of the fluorene derivatives studied in different solvents. Fluorene derivatives concentration 1.0 x 10^{-6} M

Compound	***Cyclohexene***	***Ethanol***	***Acetonitrile***	***DMSO***	***Water***
**1**	304, 312 (260)	305, 313 (290)	305, 315, 340 (sh) (257)	340 (260)	305, 313, 425 (sh) (254)
**2**	---	305, 315, 340 (254)	340 (sh), 435 (258)	340 (266)	425 (255)
**3**	336, 350, 429 (310)	336, 352, 425 (305)	336, 352, (302)	336 (308)	348 (296)
**4**	336, 353, 400, 429, 450 (305)	337, 352, 409, 429 (305)	340, 354 (302)	335, 354 (sh) (305)	353 (284)

sh: shoulder
